# Misdiagnosis of obstetrical cases and the clinical and cost consequences to patients: a cross-sectional study of urban providers in the Philippines

**DOI:** 10.3402/gha.v9.32672

**Published:** 2016-12-15

**Authors:** Riti Shimkhada, Orville Solon, Diana Tamondong-Lachica, John W. Peabody

**Affiliations:** 1 QURE Healthcare, San Francisco, CA, USA; 2 School of Economics, University of Philippines, Quezon City, Philippines; 3 Global Health Sciences, University of California, San Francisco, CA, USA

**Keywords:** misdiagnosis, health outcomes, health costs, Philippines, quality of care, practice variability, obstetrics

## Abstract

**Background:**

Misdiagnosis may be a significant and under-recognized quality of care problem. In birthing facilities located in anurban Philippine setting, we investigated the diagnostic accuracy for three obstetric conditions: cephalopelvic disproportion (CPD), post-partum hemorrhage (PPH), and pre-eclampsia.

**Design:**

Identical simulated cases were used to measure diagnostic accuracy for every provider (*n*=103). We linked misdiagnosis – identified by the simulated cases – to obstetrical complications of the patients at the participating facilities. Patient-level data on health outcomes and costs were obtained from medical records and follow-home in-person interviews.

**Results:**

The prevalence of misdiagnosis among obstetric providers was 29.8% overall, 25% for CPD, 33% for PPH, and 31% for pre-eclampsia. Linking provider decision-making to patients, we found those who misdiagnosed the simulated cases were more likely to have patients with a complication (OR 2.96; 95% CI 1.39–3.77) compared with those who did not misdiagnose. Complicated patients were significantly less likely to be referred to a hospital immediately, were more likely to be readmitted to a hospital after delivery, had significantly higher medical costs, and lost more income than non-complicated patients.

**Conclusion:**

Diagnosis is arguably the most important task a clinician performs because it determines the subsequent course of evaluation and treatment, with the direct and indirect costs of diagnostic error, placing large financial burdens on the patient.

## Introduction

Arguably, the most important clinical judgment made by a provider is labeling a patient with a diagnosis. Diagnosis is the foundation for all decisions about treatment and a key determinant of a successful outcome. Recent reports on poor care quality indicate that medical misdiagnosis, or diagnostic error, is a significant problem that has not been fully recognized (1–5). The landmark US Institute of Medicine (IOM) report brought to light a worrying statistic: medical errors, likely, kill more people than traffic accidents in the United States (6). This IOM study was one in a series of studies over the past few decades worldwide, showing the variation in quality of care. Despite an urgency to study and reduce diagnostic errors, this area has remained an under-emphasized and under-studied area of quality research.

Early work on quality improvement has focused on the lack of structural inputs. This work then gave way to models that started to look at care processes, which are more proximal determinants of health outcomes (6). Today, investigations into quality of care are increasingly conceptualizing care as patient-centered and/or timely with efficient delivery of *effective* services (7). This has shifted thinking to the idea that poor quality occurs when providers are unable to process information that they have gathered and then translate this information into proper patient treatment (8). However, this presumes that the data gathering and processing performed by providers is adequate, but this is not always the case, especially with medical misdiagnosis.

Misdiagnosis is a significant quality of care shortcoming with worrisome, albeit poorly understood, consequences. For example, a US study, reported that 5% of adults are misdiagnosed during outpatient visits, and about 50% of these errors could prove to be harmful for the patient (2). Misdiagnosis in breast cancer is reportedly over 20% in some cases (9). In low- and middle-income countries, the rates of misdiagnosis are likely more pronounced and the consequences more concerning (10). An observational study of primary care providers in rural China found the misdiagnosis rate was 74%, with clinicians consequently providing unnecessary or harmful medicine to 64% of their patients (11). In India, alarmingly low rates of diagnosis appear to be an issue, with only one-third even articulating a diagnosis in standardized patients, regardless of whether the diagnosis was correct or incorrect (12). Equally alarming was the poor adherence to treatment guidelines and frequent use of harmful or unnecessary drugs, with 62% of asthma cases being prescribed wrong or harmful treatment and 69% of patients with symptoms of unstable angina not given the proper medications. In addition, only 12% of standardized patients whose child had symptoms of dysentery were asked to give the child oral rehydration therapy. Findings on diagnostic errors such as these, as well as the consequences to treatment, come from all over the world and occur in all types of care settings. This suggests the need for a fundamental shift in conceptualizing quality of care deficiencies to include misdiagnosis.

Real-world practicalities, however, make investigating misdiagnoses a substantial challenge. Methodologic problems include the aggregation of enough patients with the same diagnosis to overcome the unobserved (and unrecorded) case mix variation; the legitimate disagreements on reference standards for practice; the continuous reliance on recorded retrospective data; and the challenges of measuring a clinician's cognitive thought processes. Perhaps the biggest methodological challenge, however, is that any review of diagnostic accuracy has to start with some agreement on what the diagnosis really is for that case. Short of re-examining the patient with a group of experts at the time the patient is seen, evaluating for correct diagnoses cannot be done easily.

To overcome these methodological challenges, we needed a tool that allows insight into how different providers handle equivalent clinical encounters and can be deployed in a number of settings at a low cost. To our knowledge, Clinical Performance and Value (CPV^®^) vignettes are the only validated means by which providers could be so measured (13–16). They have been widely used in the United States (17–22) and in low- and middle-income settings (14, 23–25). These case vignettes, or simulated cases, when combined with actual patient data offer some advantages for evaluating the presence, causes, and consequences of misdiagnosis. The CPV vignettes used in this study were designed specifically to simulate various obstetric complications and to guide providers through a patient encounter. Each provider in the study took all available vignettes to ascertain their ability to correctly diagnose a patient, and the results allow for the direct comparison of provider actions.

With a naturally time-bound clinical course, obstetrical care offers an ideal opportunity to use the CPVs to evaluate the clinical complications and economic costs of misdiagnosis. By combining vignette data on misdiagnosis with patient record data and post-discharge interviews, it is possible to quantify misdiagnosis rates and their consequences among obstetricians and midwives. The ability to immediately recognize (diagnose) and appropriately respond to (treat) a pressing obstetric complication is a key skill that providers must possess.

The objective of this study was to examine the overall rate of misdiagnosis and quality of care among obstetric providers in an urban middle-income country setting. We also wanted to better understand and classify the types of errors that led to misdiagnosis. By doing so, we hope to better understand the types of errors that lead to misdiagnosis. As part of a more patient-focused outcome, the study also examined the clinical and economic consequences of misdiagnosis by reviewing both patient medical charts and a follow-home survey.

## Methods

Completed in an urban Philippine setting, this study investigated the diagnosis of three common obstetric conditions using the following three CPV vignette case types: cephalopelvic disproportion (CPD), post-partum hemorrhage (PPH), and pre-eclampsia (Pre-Ec).

Misdiagnosis was ascertained from the vignettes by comparing the provider's response to the diagnosis of the particular vignette. Every vignette case type had a primary diagnosis, a secondary diagnosis, and indicated the severity of the primary diagnosis. To link misdiagnosis to patient-level data, we used the CPV data for all study providers and examined the medical charts of patients with obstetric complications at each participating provider's health facility. In addition to the patient chart data, in-person patient interviews were conducted to obtain additional information on health outcomes and costs during and after childbirth. Consent was obtained for participation in this study from all participating providers and patients.

### Study site

The study site, Quezon City, is located in Metro Manila of the Philippines which accounts for 23% of the total Metro Manila population and houses almost 20,000 persons per square kilometer. The city was selected for the study due to its large numbers of public and private health facilities, which include 62 hospitals, 65 health centers, and 7 lying-in clinics (26). The perceived openness of health authorities and local government officials to health-related research and improving patient care also contributed to its selection as the study site. For inclusion in this study, we contacted all birthing facilities that met the following criteria: 1) a physical infrastructure or place located outside of the patient's home; 2) staffed by skilled birth attendants specifically physicians, nurses, and midwives; 3) located in Quezon City; and 4) neither a hospital nor based in a hospital.

### Data sources

The data frame came from the Q@B study, carried out in 77 birthing facilities in Quezon City out of a total of 108 eligible facilities; there were 31 refusals. From these 77 birthing facilities, we were given a roster of providers and patients’ complete medical charts identified from June to November 2013. To determine the occurrence of an adverse event among rostered patients, we reviewed the medical records and conducted maternal exit surveys.

Within 6 months of delivery, we administered a follow-home survey to all of the mothers in our study. We surveyed for patient-reported outcomes and health service utilization along with detailed information on costs of care, both direct (e.g. supplies, medicines, diagnostic tests, doctor's/midwife's fee, facility fee, food, transportation, and other miscellaneous fees during initial admission and readmission for complications, if any) and indirect (e.g. costs of in-home care and loss of work) incurred between childbirth and 8 weeks post-partum.

A risk profile for complications was determined for every patient in the study. From these data, 33 patients with complications were identified. These patients were linked to their providers, 24 in total.

We defined complications as any one of the following: fever, abnormal vaginal discharge, urinary incontinence, excessive bleeding, perineal tears, high blood pressure, seizures, jaundice, prolonged labor, inability to deliver vaginally including caesarean delivery, and need for blood transfusion. Clinical findings during the peri-partal period that were less specific and less likely to be birth-related were excluded, such as headache, blurring of vision, muscle or joint pains, and nasal congestion.

Using the same patient rosters of the same 77 participating birthing facilities, we matched patients with complications to patients without complications, based on their risk profile. We calculated a sample size for patients without complications based on a *p*<0.05 level of significance and 80% power and the standard deviation estimated from the Q@B data. This rendered a without complication sample size requirement of 92 patients, who were linked to 79 unique providers.

All 103 providers (24 that had patients with complications and 79 that did not) were asked to take the vignettes.

### CPV vignettes

CPV vignettes are simulated patient cases designed to mimic the doctor–patient clinical interaction. CPVs have been validated as a quality measure of a physician's ability to evaluate, diagnose, and treat specific diseases and conditions (13, 14, 27, 28). The CPVs are patient cases given electronically to a group of providers simultaneously. They are open ended and comprehensively assess a provider's clinical practice in five domains: 1) taking a medical history, 2) performing a physical exam, 3) ordering tests, 4) making a diagnosis, and 5) prescribing a treatment plan. Each completed vignette was independently scored by two obstetrical (midwives or physicians) abstractors; any noted discrepancies were reconciled by a third physician abstractor. Because every provider took care of the same cases (i.e. there was no case-mix adjustment), we were able to determine where diagnostic errors are made, what treatments were employed, and the costs of those evaluations and treatments. The CPV cases were scored based on standard international obstetric clinical guidelines, and scores ranging from 0 to 100% were calculated by determining the number of items correctly answered and dividing by the total number of items for each domain and over all five domains (total).

The three vignettes used in this study involved simulated obstetric patients who had signs and symptoms of an obstetrical complication – Pre-Ec, CPD, and PPH. Each case type was designed to ascertain whether a provider was able to correctly identify and subsequently treat the complication (see Supplementary file for survey instruments used).

Between January and April 2014, we collected CPV data on the quality of care, which included data on whether a correct diagnosis had been made on the simulated cases. The 103 providers each completed three maternal CPV vignettes (a total of 309 vignettes were completed). Each of the three vignettes tested a specific complication related to childbirth: one for Pre-Ec, one for CPD, and one for PPH from uterine atony. Responses to the vignettes were scored against explicit criteria allowing us to determine if the providers took the proper steps to ascertain the risk-profile of the mother, make the correct diagnosis, and identify that a complication occurred.

### Conceptual framework

**Fig. 1:**
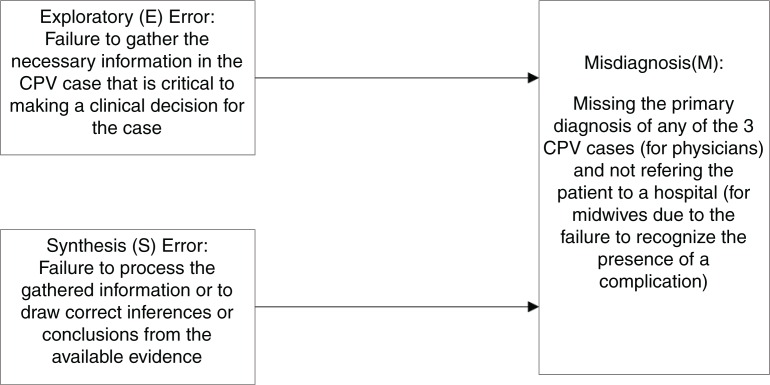
Framework for conceptualizing misdiagnosis.

Our conceptual framework proposes that the items in the CPV vignettes can be used to identify two different types of errors that lead to misdiagnosis on the part of a clinical decision-maker (Fig. 1). The first is an exploratory (E) error, which is the failure to gather the necessary clinical information. It is assumed in this framework (and in the way the CPV vignettes are written) that being able to gather the necessary information reduces diagnostic errors; for example, a failure to ask about the past obstetric history (e.g. gravida and parity) would limit the ability to diagnose a patient as high risk. The second type of error is a synthesis (S) error, which occurs when a provider is unable to synthesize the gathered information and fails to draw correct inferences or make a diagnosis from the available evidence; for example, not ordering a hematocrit when a patient is pale or tachycardic. Table 1 provides an example of how the CPV vignette presents a CPD case and shows how each item is categorized into either E or S errors. We hypothesized that having more exploratory errors or synthesis errors on the CPV increases the likelihood that a provider will make an incorrect diagnosis on the CPV.

**Table 1:** CPV items and the different error types: the cephalopelvic disproportion vignette

	Error type
History of present illness	
Asks about gravidity and parity	E
Asks about age of gestation (LMP or early ultrasound)	E
Asks about bloody show or vaginal bleeding	E
Asks about rupture of bag of waters	E
Asks about onset, frequency, and quality of contractions	E
Asks about good fetal movement or change in fetal movement	E
Asks about number and findings of prenatal checkup including the last one	E
Asks about results of previous work-up or laboratories/imaging	E
Asks about abnormal symptoms (fever, cough and colds, loss of consciousness, abdominal pain, dyspnea, headache, dysuria, foul-smelling vaginal discharge, and edema)	E
Past medical and obstetric history	
Asks about manner of delivery of previous pregnancies	S
Asks about complications of previous pregnancies (gestational hypertension/pre-eclampsia, gestational diabetes, UTI, preterm labor, and post-partum hemorrhage)	E
Asks about birthweight of previous deliveries	S
Asks about comorbidities (hypertension, diabetes, thyroid disorder, asthma, and infections)	E
Asks about previous surgeries	E
Asks about use of medications including recent vaccinations	E
Asks about allergies to food and drugs	E
Asks about regularity of menses prior to pregnancy and/or use of contraceptive methods	E
Family medical and social history	
Asks about family history of anemia, bleeding disorder, hypertension, diabetes, asthma, etc.	E
Asks about relationship status (married/live-in/single)	E
Asks about current employment, highest educational attainment, insurance, and access to healthcare	E
Asks about tobacco use	E
Asks about alcohol abuse	E
Asks about use of illicit drugs	E
Asks about diet preferences and exercises	E
Physical examination	
General survey: comfortable or in distress, unconscious	E
Checks vital signs (blood pressure, heart rate, respiratory rate, and temp)	E
HEENT: pallor, jaundice, and distended neck veins	E
Lungs: breath sounds and percussion	E
Cardiac: regular rhythm and murmurs	E
Extremities: pallor, cyanosis, and edema	E
Abdomen: bowel sounds, guarding, and tenderness	E
Fundic height	E
Leopold's maneuver: fetal lie and presentation	S
Engagement of fetal head	E
Estimated fetal weight	E
Fetal heart tones (rate and location)	E
IE: confirm fetal presentation	S
Cervical dilatation	E
Cervical effacement	E
Fetal Station	E
Status of bag of waters (BOW)	E
Age of gestation	E
Gravidity and parity	E
Previous successful vaginal delivery	S
Absence of comorbidities	E
Estimated fetal weight (is it higher than first child)	S
Term pregnancy with no apparent complications	S
Diagnostic tests	
Complete blood count	S
Blood typing	S
Blood chemistry (BUN, creatinine, etc.)	S
PT/PTT	S
Chest x-ray	S
12L ECG	S
Fetal monitoring	S

LMP, last menstrual period. E-type errors are exploratory errors where the provider did not gather the correct information; S-type errors are synthesis errors where the provider did not draw proper conclusions from correct information.

### Analysis

*Study Question 1:* What is the overall rate of misdiagnosis and quality of care, as measured by the CPV vignettes, among obstetric providers?

By case types, CPV vignettes were scored to measure overall quality of care. Results were reported by averages, standard deviation, and minimum and maximum scores. CPVs were also summarized by separate domain, including the diagnosis domain again using averages and ranges, summarized in box plots. Complications were linked to their corresponding vignettes because of limited sample size. Similarly, we did not compare scores between types of provider (physician vs. midwife). We felt that the exploratory and synthetic factors that led to misdiagnosis were not case-specific and explored this relationship instead.

*Study Question 2:* Could errors in clinical thinking, identified by the CPV vignettes, be defined as errors originating from inadequate clinical data gathering (exploratory) or errors in synthesis? And was either type of error associated with more misdiagnoses?

We used a two-level random effects logistic regression with providers nested within facilities to account for possible localized provider practice similarity (e.g. standard facility practices). We examined exploratory and synthetic errors, as conceptualized in our framework (Fig. 1) and defined by items in the CPVs (Table 1), and the association between these types of errors and misdiagnosis made by the provider on the CPV (the outcome variable). For this, we controlled for CPV case type (described in Table 2) as well as provider age and whether the provider worked in a private or public facility, these provider characteristics being identified *a priori* as potential explanatory variables in the regression model. Controlling for case type allows us to determine if misdiagnosis was affected by CPV case type.

**Table 2:** Primary diagnosis items by CPV case type for physicians

CPV case type	CPV case description	Identifying characteristics of complications and severity of case	Primary diagnosis
1	Pregnancy uterine 39 weeks AOG by LMP in labor	• Obstructed labor failure of descent probably from asynclitism) • Non-reassuring fetal status/fetal compromise	Cephalopelvic disproportion
2	Hemorrhage post-delivery	• Uterine atony • Retained placental fragments • Hypovolemic shock	Post-partum hemorrhage
3	Pregnancy uterine 38 6/7 weeks AOG by LMP in labor	• BP>160/110 • High LDH • Low platelets • High AST & ALT	Pre-eclampsia

AOG, age of gestation; LMP, last menstrual period; BP, blood pressure; LDH, lactate dehydrogenase; AST, aspartate aminotransferase; ALT, alanine aminotransferase.

*Study Question 3:* After linking provider CPV vignette data with patient medical charts and the follow-home survey data, were providers who misdiagnosed simulated patients more likely to have had patients with complications and poor clinical outcomes? And were these complications associated with higher direct and indirect costs?

We first examined if providers who misdiagnosed a CPV were more likely to have had a patient complication under their care. We ran a three-level logistic regression with patients nested within providers (to account for a provider seeing similar types of patients) and providers nested within facilities (for the same reason as given for the 2-level regression). The regression model was run with complications as a function of misdiagnosis, controlling for high risk (whether the patient was determined to be high risk or not) and provider characteristics (age and public/private). High risk was defined as having any one of the following factors: aged <19 or >35 years; pregnancy BMI of <19 or >25 kg/m^2^; height <5 ft; history of stillbirth, miscarriages, abortions, preterm labor/delivery, ectopic pregnancy, or profuse bleeding 6 months after delivery; >5 pregnancies; third trimester bleeding during the most recent pregnancy; diagnosed by the physician with hypertension, diabetes, and other comorbidities (e.g. bronchial asthma, heart disease, thyroid disease, sexually transmitted infection, and seizure disorder) prior to the most recent pregnancy; and smoking, drinking, or other drugs during pregnancy.

From our follow-home survey, we examined whether poor patient-reported outcomes and costs of care were positively associated with complications. Student's *t*-tests were used to compare both the clinical and the direct and indirect monetary costs (number of months not worked and average monthly forgone income) between the groups of patients who had complications versus those who did not.

Poor outcomes were defined as when mothers reported that they either stayed in the birthing facility and were not referred immediately, needed a post-discharge consultation for ongoing problems, had a post-discharge hospital admission, or the newborn child needed follow-on care. All direct and indirect costs of care were ascertained from the mother at the post-partum follow-home survey with the answers provided in the survey as the sole basis for the cost analyses. Direct costs were defined as all medical and non-medical expenses related to the case, and indirect costs included the forgone income as consequence of undergoing medical care of maternal cases with and without complications.

All analyses were completed using STATA 13.0. Statistical significance was ascertained at the *p*<0.05 level.

## Results

In total, 125 patients (both with and without complications) were included in this study, and these patients saw 103 unique providers, who provided services in 77 facilities in Quezon City. Of the 103 providers, only 22 had more than one patient in the study, with none having more than two included patients, and of the 77 facilities, only 11 had multiple providers included in the study.

### Overall quality of care and rate of misdiagnosis (Question 1)

**Fig. 2:**
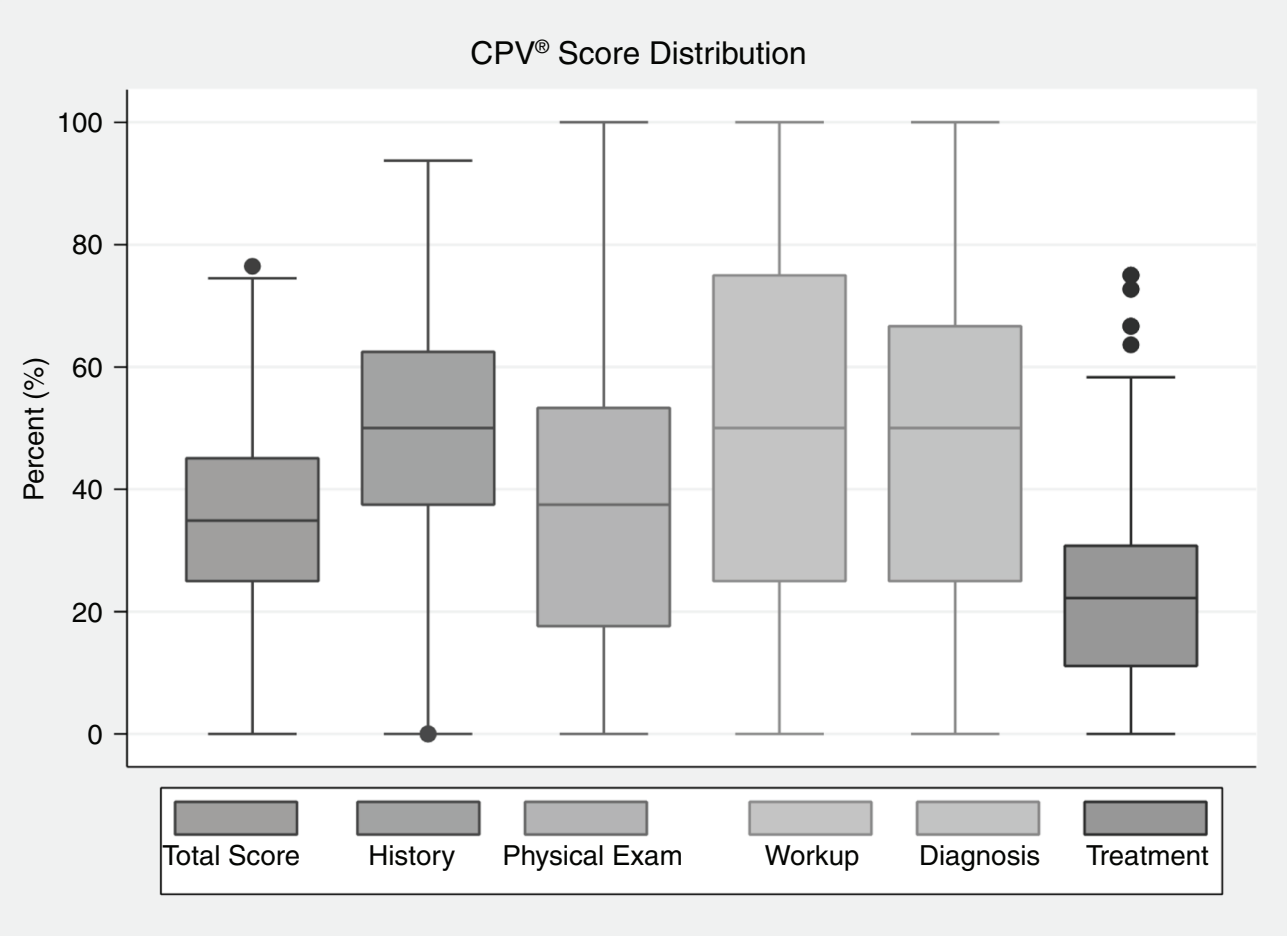
Distribution of CPV scores (*n*=309), overall and domain for 103 providers (physicians and midwives).

There were 94 midwives and 9 physicians in this study. The average age of provider was 42 years and 77% were private providers. The average overall CPV score for all three cases for the entire sample of providers was 43.4 (standard deviation 15.2). The most notable observation is that there was a large variation in overall practice and in each of the five domains. Scores ranged from 100% correct in the physical examination and work up and even diagnosis to 0%, indicating a wide range of skills (see Fig. 2). By domain, the treatment score was the lowest quality score (mean 22.9; standard deviation 16.0). The aggregate rate of misdiagnosis was about 30% for all three cases combined. We looked at several aspects of misdiagnosis: primary diagnosis, secondary diagnosis, and the ability to recognize severity score. The misdiagnosis rate varied by case but was generally poor regardless of the clinical condition (shown by case type in Table 3). About 75% of providers missed the secondary diagnosis and 74% missed questions regarding the severity of the patient's condition.

**Table 3:** Misdiagnosis among OB providers by CPV case type

CPV case^a^	% of providers who misdiagnosed cases^b^
Total misdiagnosis rate	29.8
Individual case types	
Cephalopelvic disproportion	25.2
Post-partum hemorrhage	33.0
Pre-eclampsia	31.0

CPV, Clinical Performance and Value.

a Total misdiagnosis rate based on 309 CPV vignettes, individual case type misdiagnosis based on 103 vignettes for each case type;

b misdiagnosis defined as missing primary diagnosis by the physician who took the CPV vignette and non-referral of the patient by the midwife who took the CPV vignette.

### Impact of exploratory, synthesis, and judgment errors on misdiagnosis (Question 2)

To determine the provider-level predictors of misdiagnosis, we estimated a two-level random effects logistic regression with providers nested within facilities (Table 4). The model includes error type in the CPV, CPV case taken by the provider, provider age, and provider employment in a public versus private facility. The model shows that committing both E (OR=1.91, 95% CI 1.31–2.99) and S (OR=2.24, 95% CI 1.03–3.69) type errors are statistically significant predictors of misdiagnosis compared with not making an error (*p*<0.05). There were no differences in this association by type of case, either. The only provider characteristic associated with misdiagnosis is age, with misdiagnosis decreasing with age of provider (OR=0.89, 95% CI 0.69–0.99).

**Table 4:** Predictors of misdiagnosis, logistic regression model (*n*=309)

	Odds ratio (95% CI)	*p*
Error type		
No error	Ref	
Exploratory	1.91 (1.31, 2.99)	0.048
Synthesis	2.24 (1.03, 3.69)	0.049
CPV type		
Cephalopelvic disproportion	Ref	
Post-partum hemorrhage	1.33 (0.87, 2.91)	0.896
Pre-eclampsia	1.54 (0.80, 3.11)	0.712
Provider characteristics		
Private	Ref	
Public	1.39 (0.99, 1.62)	0.293
Age (continuous)	0.88 (0.68, 0.99)	0.003

CPV, Clinical Performance and Value.

### Link between misdiagnosis and complications (Question 3a)

The most common complications, necessitating referral to higher level facilities, were an emergency C-section (*n*=18), fetal distress (*n*=10), hypertension (*n*=6), and bleeding requiring blood transfusion (*n*=6). In total, there were 70 such complications, but of these, only 33 could be directly linked to a specific provider. The other 37 patients whose complications could not be directly linked were excluded from the study.

To examine the association between provider misdiagnosis, as measured by the CPVs, and misdiagnosis, we performed a three-level random effects logistic regression. Complications were determined by the presence of complications in the patient record. We found that providers who misdiagnosed the CPV were significantly more likely (OR 2.97, 95% CI 1.41–3.32) to have patients with a complication compared with providers who did not misdiagnose (see Table 5). High-risk pregnancies, as expected, had an association (OR 2.38, 95% CI 1.23–4.72) with the presence of a complication. The model controlled for provider characteristics and CPV type, but there were no statistically significant findings between these variables and the presence of a complication.

**Table 5:** Association between provider misdiagnosis in the CPV and presence of any patient complication in the patient (outcome), logistic regression model (*n*=309)

	Odds ratio (95% CI)	*p*
Misdiagnosis		
No	Ref	
Yes (primary)	2.97 (1.41, 3.32)	0.05
CPV type		
Cephalopelvic disproportion	Ref	
Post-partum hemorrhage	1.13 (0.83, 2.31)	0.36
Pre-eclampsia	1.02 (0.84, 2.58)	0.57
High-risk patient	2.38 (1.23, 4.72)	0.04
Provider characteristics		
Private	Ref	
Public	0.91 (0.82, 1.21)	0.42
Age (continuous)	1.03 (0.79, 1.09)	0.67

CPV, Clinical Performance and Value.

### Clinical and monetary costs of complications (Question 3b)

The follow-home survey allowed examination of additional patient-reported outcomes not available in the exit or medical chart, such as post-discharge outcomes, direct costs of care, and lost income (see Table 6). We found that the 33 patients with complications identified from the charts, were less likely to be referred to a hospital immediately upon diagnosis (*p*=0.001). In addition, the patients with complications were more likely to have required hospital readmission after being discharged at birth (*p*=0.021). We did not find a statistically significant difference in the care needs for the newborn child.

**Table 6:** Patient outcomes, costs, and income forgone for complication versus non-complications, as reported in the follow-home survey

	Complications (*n*=33)	Non-complications (*n*=37)	*p*
Follow-home outcomes			
Not referred immediately from the birthing facility to hospital	72.7%	23.7%	0.001
Had post-discharge consult	9.1	8.5	0.198
Had post-discharge hospital admission	9.1	0.0	0.021
Child needed follow-on care	36.4	37.3	0.88
Cost of care (in PhP)			
Total direct expenses	25,969	12,958	0.015
For those who stayed in birthing facility for delivery	7,305	6,744	0.441
For those who gave birth in hospital	17,343	4,973	0.002
For those who were admitted post-discharge	818	0	0.021
Newborn use of care	502	1,241	0.271
Work/income forgone			
Months since discharged	6.2	4.5	0.54
Equivalent months worked	1.6	3.1	0.48
Equivalent months not worked	4.6	1.4	0.039
Average monthly income forgone (PhP)	67,100	17,846	0.034

The direct costs of care for patients with complications were significantly higher than for patients without complications (25,969 vs. 12,958 PhP; *p*=0.015); this disparity was found among women who gave birth in the hospital, but not for those who stayed in the birthing facility (see Table 6). Only the patients with complications incurred costs post-discharge stemming from post-discharge hospital readmissions (i.e. non-complications were not readmitted after leaving the hospital).

To examine the reported costs of care and lost wages (indirect costs) due to childbirth and recovery from childbirth, we compared the number of months worked/not worked and the average monthly forgone income for complications versus non-complications. We observed that the number of months not worked after having a baby were significantly higher for patients with complications (4.6 months vs. 1.4 months; *p*=0.04), and the average income forgone was significantly higher (67,100 PhP/month vs. 17,846 PhP/month; *p*=0.03) among those with complications compared with those without complications (see Table 6).

## Discussion

This study demonstrates the importance of correct diagnoses in preventing poor clinical outcomes and economic losses to the patient. By using CPV vignettes to measure misdiagnosis on standardized patients and patient record data, we were able to measure the care given by 103 midwives and physicians providing obstetric care at birthing facilities in Quezon City. This study shows the challenges related to obstetrical providers in providing correct diagnoses for patients, both in the birthing facility and in the vignettes. We found that those who incorrectly identified the diagnosis on the CPV vignettes were nearly three times more likely to have patients who had complications during childbirth. These complications, in turn, led to more negative patient outcomes, such as two times the out-of-pocket costs, three times the delay in hospital referral, and nearly four times the wage loss.

Making a diagnosis is arguably the single most important early task a clinician performs during a clinical encounter. The diagnosis determines the subsequent course of evaluation and treatment (29), with a diagnostic error leading to unnecessary evaluations and treatments under the best circumstances or harmful tests and toxic treatments under the worst. As seen in this study, these errors are costly financially, and the result in potential treatment delays put patients at risk (2). Recent research shows that diagnostic errors do in fact lead to severe consequences and are an unrecognized area of preventable morbidity and mortality (3–5, 29).

Obstetrics, in particular, remains a challenge to many low- and middle-income countries. While maternal mortality ratio dropped by 45% globally between 1990 and 2013, from 380 to 210 deaths per 100,000 live births, this fell short of targets to reduce the maternal mortality ratio by three quarters by 2015 (30). The quality problem we address here is summed up in these poor statistics and this unfortunate fact: most of the conditions leading to death were completely preventable, and death should have been completely avoided if there was a minimum level of quality of care provided for those who did actually encounter healthcare. While there are well-known healthcare solutions for the prevention and handling of medical complications that lead to death, many healthcare providers are unable to provide proper care, often because they simply lack the clinical skill. In the case of many maternal deaths, simple uterotonic commodities and magnesium sulfate can prevent and/or manage common complications such as bleeding during childbirth and hypertensive disorders caused by pregnancy.

While our vignette/simulation methodology offers advantages as a quality measure of diagnostic errors over studies that rely on chart review or administrative data, there are important limitations in this particular study. The biggest limitation is having a small sample size with too few physician providers to make subgroup comparisons with midwives. There are likely different clinical thought processes between midwives, who are trained to recognize complications and then immediately refer a patient, and physicians, who must also be able to treat upon recognizing a complication. From our other work in evaluating quality, we suspect that there are also differences in clinical reasoning by case type that need to be explored (18). Another potential limitation in this study is recall bias, that is, relying on the recollection of patients regarding their care utilization and the cost of the care. While it is likely that any readmission or major obstetric complication would be accurately recalled in a 6-month or less time frame, recalling the cost of that care will be less reliable.

The important and significant problem of misdiagnosis, to us, compels the need for measuring and evaluating the quality of clinical care, especially in low- and middle-income countries, and simulated cases appear to be useful in evaluating the rate of misdiagnosis and the likelihood that clinically worrisome and economically costly complications are occurring among the patients that providers are seeing. However, measurement and accountability require a policy change to fund affordable programs, such as the vignette-based method, that regularly assess knowledge and capability of health professionals and provide them with engagement, instruction, and supportive feedback. The goal of these (often large scale) programs is to improve the quality of care through education and engagement.

Specifically, to reduce the rates of medical misdiagnosis, we recommend a collective effort between the Department of Health who designs and administers policies and standards for health facilities, the Professional Regulations Commission who has the mandate of licensing and regulation of health professionals, the specialty societies in assisting providers make appropriate and timely decisions around referrals, and the National Health Insurance (PhilHealth).

Future work should evaluate if serial measurements of diagnostic accuracy, when coupled with provider benchmarking and feedback, will change provider's clinical skill and significantly improve health status, including both clinical outcomes and costs.

## Conclusions

The prevalence of misdiagnosis in this study of urban obstetric providers was notably high: 29.8% overall, and relatively consistent across the three diagnoses: 25.2% for CPD, 33% for PPH, and 31% for Pre-Ec. Errors in synthesis and judgment increased the chance of making a misdiagnosis and contributed to misdiagnoses about equally. Providers who misdiagnosed on the standardized vignette cases were found to be more likely to have patients with a complication than providers who did not misdiagnose. These complicated patients were less likely to have been referred immediately, were more likely to have been readmitted to a hospital after discharge, had significantly higher medical costs, and had higher lost income than non-complicated patients. The study here and other reports suggest misdiagnosis is a significant quality of care failing with worrisome clinical and economic consequences, thus making misdiagnosis a topic of high interest for further study and policy action globally.

## Supplementary Material

Misdiagnosis of obstetrical cases and the clinical and cost consequences to patients: a cross-sectional study of urban providers in the Philippines

## Paper context

Clinical diagnosis is likely the most important judgment made by doctors and is almost certainly under-recognized as a quality of care problem across the globe. This is particularly true in obstetrical care delivered in developing countries, where maternal mortality remains unacceptably high. This study provides objective data on the high prevalence of misdiagnosis among pregnant women with three common complications and estimates the associated direct and indirect patient costs in an urban setting in the Philippines.

## References

[1] Manski CF (2013). Diagnostic testing and treatment under ambiguity: using decision analysis to inform clinical practice. Proc Natl Acad Sci.

[2] Singh H, Meyer AN, Thomas EJ (2014). The frequency of diagnostic errors in outpatient care: estimations from three large observational studies involving US adult populations. BMJ Qual Saf.

[3] Singh H, Sittig DF (2015). Advancing the science of measurement of diagnostic errors in healthcare: the Safer Dx framework. BMJ Qual Saf.

[4] Zwaan L, de Bruijne M, Wagner C, Thijs A, Smits M, van der Wal G (2010). Patient record review of the incidence, consequences, and causes of diagnostic adverse events. Arch Intern Med.

[5] Zwaan L, Thijs A, Wagner C, van der Wal G, Timmermans DR (2012). Relating faults in diagnostic reasoning with diagnostic errors and patient harm. Acad Med.

[6] Institute of Medicine (2002). Crossing the quality chasm: a new health system for the 21st century.

[7] Cassel CK, Conway PH, Delbanco SF, Jha AK, Saunders RS, Lee TH (2014). Getting more performance from performance measurement. N Engl J Med.

[8] Mostofian F, Ruban C, Simunovic N, Bhandari M (2015). Changing physician behavior: what works?. Am J Manag Care.

[9] Elmore JG, Longton GM, Carney PA, Geller BM, Onega T, Tosteson AN (2015). Diagnostic concordance among pathologists interpreting breast biopsy specimens. JAMA.

[10] Scott KW, Jha AK (2014). Putting quality on the global health agenda. N Engl J Med.

[11] Sylvia S, Shi Y, Xue H, Tian X, Wang H, Liu Q (2015). Survey using incognito standardized patients shows poor quality care in China's rural clinics. Health Policy Plan.

[12] Das J, Holla A, Das V, Mohanan M, Tabak D, Chan B (2012). In urban and rural India, a standardized patient study showed low levels of provider training and huge quality gaps. Health Aff (Millwood).

[13] Peabody JW, Luck J, Glassman P, Dresselhaus TR, Lee M (2000). Comparison of vignettes, standardized patients, and chart abstraction: a prospective validation study of 3 methods for measuring quality. JAMA.

[14] Peabody JW, Luck J, Glassman P, Jain S, Hansen J, Spell M (2004). Measuring the quality of physician practice by using clinical vignettes: a prospective validation study. Ann Intern Med.

[15] Peabody JW, Tozija F, Munoz JA, Nordyke RJ, Luck J (2004). Using vignettes to compare the quality of clinical care variation in economically divergent countries. Health Serv Res.

[16] Converse L, Barrett K, Rich E, Reschovsky J (2015). Methods of observing variations in physicians’ decisions: the opportunities of clinical vignettes. J Gen Intern Med.

[17] Peabody JW, Strand V, Shimkhada R, Lee R, Chernoff D (2013). Impact of rheumatoid arthritis disease activity test on clinical practice. PLoS One.

[18] DeMaria L, Acelajado MC, Luck J, Ta H, Chernoff D, Florentino J (2014). Variations and practice in the care of patients with rheumatoid arthritis: quality and cost of care. J Clin Rheumatol.

[19] Peabody JW, Shimkhada R, Tong KB, Zubiller MB (2014). New thinking on clinical utility: hard lessons for molecular diagnostics. Am J Manag Care.

[20] Peabody JW, Huang X, Shimkhada R, Rosenthal M (2015). Managing specialty care in an era of heightened accountability: emphasizing quality and accelerating savings. Am J Manag Care.

[21] Fields KK, Soliman HH, Friedman EL, Lee RV, Acelajado MC, Tamondong-Lachica D (2013). Measuring clinical pathway compliance using a simulated patient approach with Clinical Performance and Value (CPV) vignettes. J Clin Oncol.

[22] Letson DD, Fields KK, Hammon DK, Lee RV, Peabody JW, List AF (2013). A provider-payor approach for determining value in the health reform era: early reports on the M-QURE initiative. J Clin Oncol.

[23] Peabody JW, Shimkhada R, Quimbo S, Solon O, Javier X, McCulloch C (2014). The impact of performance incentives on child health outcomes: results from a cluster randomized controlled trial in the Philippines. Health Policy Plan.

[24] Peabody JW, Luck J, DeMaria L, Menon R (2014). Quality of care and health status in Ukraine. BMC Health Serv Res.

[25] Quimbo S, Wagner N, Florentino J, Solon O, Peabody J (2016). Do health reforms to improve quality have long-term effects? Results of a follow-up on a randomized policy experiment in the Philippines. Health Econ.

[26] Local Government of Quezon City Facts and figures 2015.

[27] Dresselhaus TR, Peabody JW, Lee M, Wang MM, Luck J (2000). Measuring compliance with preventive care guidelines: standardized patients, clinical vignettes, and the medical record. J Gen Intern Med.

[28] Dresselhaus TR, Peabody JW, Luck J, Bertenthal D (2004). An evaluation of vignettes for predicting variation in the quality of preventive care. J Gen Intern Med.

[29] Zwaan L, Schiff GD, Singh H (2013). Advancing the research agenda for diagnostic error reduction. BMJ Qual Saf.

[30] World Health Organization (WHO) Maternal mortality.

