# Supplementary material for: Misdiagnosis of obstetrical cases and the clinical and cost consequences to patients: a cross-sectional study of urban providers in the Philippines
Source: Glob Health Action. 2016 Dec 15;9:10.3402/gha.v9.32672. doi: 10.3402/gha.v9.32672 (PMC5161800; doi:10.3402/gha.v9.32672)
Supplement: Misdiagnosis of obstetrical cases and the clinical and cost consequences to patients: a cross-sectional study of urban providers in the Philippines [file GHA-9-32672-s001.pdf]

Supplementary File: Survey Instruments for the CPV® Vignettes.

CPV #1: Cephalopelvic Disproportion

FIRST DO NO HARM
CLINICAL PERFORMANCE VIGNETTE #1
February 2014

A. SAMPLING INFORMATION

A.1.	Date completed:	___ ___ /___ ___ /___ ___ (mm dd yy)	
A.2.	Time started:	__ __ : __ __ H	
A.3.	Time completed:	__ __ : __ __ H	
A.4.	Round:	0 1	
A.5.	City:	Quezon City	
A.6.	District:	_________________	
A.7.	Facility ID #:	____ ____ (Reg) ____ ____ (Prov) ____ ____ (Dist) ____ ____ ____ (Facility Code)	
A.8.	Provider ID #:	____ ____ (Reg) ____ ____ (Prov) ____ ____ (Dist) ______ (Pub/Pvt) ___ ___ ___ (Facility Code) ___ ___ ___ (Provider Code)	
A.9.	Provider type:	General Practitioner..	1
		Obstetrician-Gynecologist..	2
		Nurse-midwife..	3
		Nurse...	4
		Midwife...	5
		TBA/ *hilot*..	6
		Other, specify _____________...	7
A.10.	Age from last birthday:	___ ___	
A.11.	Sex:	Male..	1
		Female..	0
A.12.	Contact number *(landline and/or mobile)*:	*Landline number:* 0 ___ ___ (Area code) - ___ ___ ___ ___ ___ ___ ___	*Mobile number:* 0 9 ___ ___ - ___ ___ ___ ___ ___ ___ ___

CPV Administered by: ______________________________________
Signature over printed name

Admin. Code

SUPERVISOR		SURVEY LEADER	
Printed name and signature	*Date*	*Printed name and signature*	*Date*

Provider ID # ___ ___ (*Reg*) ___ ___ (*Prov*) ___ ___ (*Dist*) ___ (*Pub/Pvt*) ___ ___ ___ (*Facility Code*) ___ ___ ___ (*Provider Code*)

B. CONSENT

Dear Respondent,

We are from QURE Healthcare, a research group working with the Philippine Department of Health. With their endorsement, we are studying the clinical and economic consequences of referrals and outcomes, on parturient mothers in Quezon City. The results of this study, which are generalizable to other low to middle income countries, will be used to increase knowledge, build collaborative efforts locally and disseminate these findings with the World Bank, which is looking to fund policy initiatives to improve quality of care.

The information that you will provide today will be treated with utmost confidentiality. The data will be used for research purposes only. Your name or address and other personal information will be deleted from the questionnaire and only a code or number will connect your name with your answers.

The vignette takes approximately 30 minutes to complete. Your participation is voluntary. If you have any questions, you can ask me or can contact our research leads in the Philippines namely Dr Diana Tamondong-Lachica at 0922-8721686 and Ms Jenifer Tiu at 0928-5000122 or at (02) 9279686 loc 322.

Your signature indicates that you understood the purpose and mechanics of this study and that you are willing to participate.

Signature over printed name of respondent

Date: __ __ /__ __ /__ __
mm dd yy

Signature over printed name of enumerator

Date: __ __ /__ __ /__ __
mm dd yy

Did provider:	Consent and sign?	1
	Consent but not sign?	2
	Refuse to participate?	3

C. INSTRUCTIONS FOR COMPLETING THE VIGNETTE OR SIMULATED CASE:

Please consider the following case. It is meant to be typical of a case you would encounter in your practice.

Please bear in mind the following assumptions / conditions while answering the case:

1. Please assume that you are the only health professional handling the case;
2. Please assume that the setting and resources available of the case are identical to where you are currently / usually working;
3. Please handle the case as you would an actual case;
4. Expected answers that are not documented (written down) are considered not done / not thought of by the respondent.

The case takes approximately 30-35 minutes to complete. It is separated into parts: once you have read the clinical material on the particular section, complete the question on the answer sheets provided.

Once you have answered the questions, it is important that you detach/separate the answer sheet, put them inside the envelope provided, or give them to the interviewer /evaluator before proceeding to the next part of the case.

Please continue until all pages have been completed and the answer sheets placed in the envelope.

Your responses will remain confidential and the anonymity of the collected results is assured. We appreciate your participation. If you have any questions, please let us know.

READ THIS VIGNETTE AND ANSWER **QUESTION 1** IN **4 MINUTES**

Ms Y. is a 28-year old woman, obviously in the late stage of pregnancy, reporting contractions and recent bloody vaginal discharge.

****

PLEASE ANSWER QUESTION 1 <u>ON THE NEXT PAGE</u>.

PLEASE ANSWER THE FOLLOWING QUESTION. DO NOT MOVE AHEAD TO THE NEXT PART OF THE VIGNETTE UNTIL YOU HAVE ANSWERED THE QUESTION ON THIS PAGE.

Question 1.

What are the 8-12 most important questions (information) that you want to ask from the ***patient's clinical history****? (Please be specific.)*

IMPORTANT: PLACE ANSWER SHEET IN ENVELOPE OR GIVE THE ANSWER SHEET TO AN EVALUATOR BEFORE CONTINUING.

CONTINUE READING THE VIGNETTE AND ANSWER **QUESTIONS 2 & 3** IN **4 MINUTES**

Ms Y. is a 28-year old woman, obviously in the late stage of pregnancy, reporting contractions and recent bloody vaginal discharge.

Ms. Y is a G2 P1 who comes to you today in the birthing clinic. You know from her previous visits that she is 39 weeks AOG by LMP. 4 hours ago, she noted a mucoid, blood-tinged discharge of about two teaspoons from her vagina. She did not observe any watery discharge or bright red blood per vagina. Her abdominal cramping started this morning and occurred once every 8 minutes and has steadily increased in intensity and frequency to about once every 3-4 minutes. She reports that there is no decrease in fetal movement.

She went to 4 prenatal check-ups with you and had normal blood pressures, weight and a complete blood count 1 month prior to this visit. Today, Mrs. Y. denies any fever, cough/colds, excessive weight gain, epigastric pain or dysuria in her last month of pregnancy but claims that her feet have begun to swell over the past 2 months.

****

PLEASE ANSWER THE QUESTION <u>ON THE NEXT PAGE</u>.

Provider ID # ___ ___ ___ ___ ___ ___ _____ ___ ___ ___ ___ ___ ___

Reg	Prov	Dist	Pub/Pvt	Facility Code	Provider Code

PLEASE ANSWER THE FOLLOWING QUESTION. DO NOT MOVE AHEAD TO THE NEXT PART OF THE VIGNETTE UNTIL YOU HAVE ANSWERED THE QUESTION ON THIS PAGE.

*What are the most important questions you want to know about the patient's **past medical history including OB-GYN history and associated medical illnesses**? (Please list.)*

Question 3.

*What are the 9 to 11 most important questions you want to know about the **family medical history and social history**? (Please list.)*

IMPORTANT: PLACE ANSWER SHEET IN ENVELOPE OR GIVE THE ANSWER SHEET TO AN EVALUATOR BEFORE CONTINUING.

CONTINUE READING THE VIGNETTE AND ANSWER **QUESTION 4** IN **4 MINUTES**

Ms Y. is a 28-year old woman, obviously in the late stage of pregnancy, reporting contractions and recent bloody vaginal discharge.

****

Ms. Y is a G2 P1 who comes to you today in the birthing clinic. You know from her previous visits that she is 39 weeks AOG by LMP. 4 hours ago, she noted a mucoid, blood-tinged discharge of about two teaspoons from her vagina. She did not observe any watery discharge or bright red blood per vagina. Her abdominal cramping started this morning and occurred once every 8 minutes and has steadily increased in intensity and frequency to about once every 3-4 minutes. She reports that there is no decrease in fetal movement.

She went to 4 prenatal check-ups with you and had normal blood pressures, weight and a complete blood count 1 month prior to this visit. Today, Mrs. Y. denies any fever, cough/colds, excessive weight gain, epigastric pain or dysuria in her last month of pregnancy but claims that her feet have begun to swell over the past 2 months.

****

She is not aware of any history of anemia, bleeding disorder, hypertension, diabetes or asthma in her family.

Ms. Y is currently residing with her live-in partner of 2 years. She is a recent college graduate who works in a fast food service restaurant. She previously smoked in her teens but stopped 5 years ago, she denies consuming alcohol and use of illicit substances. She admits to be picky with her food, consuming more meat than vegetables and fish.

PLEASE ANSWER THE QUESTIONS <u>ON THE NEXT PAGE</u>.

Question 4.

*What are the 6 to 8 most important elements of the **physical examination** that need to be performed on this patient?*

*(Note to examinee: **Please be specific**. For example, do not say you would "examine the knee." Instead, report what you would look for when you examine the knee, e.g. "examine the knee for redness, swelling, and point tenderness" or "evaluated knee for ligaments laxity and range of motion.")*

IMPORTANT: PLACE ANSWER SHEET IN ENVELOPE OR GIVE THE ANSWER SHEET TO AN EVALUATOR BEFORE CONTINUING.

CONTINUE READING THE VIGNETTE AND ANSWER **QUESTIONS 5** & 6 IN **5 MINUTES**

Ms Y. is a 28-year old woman, obviously in the late stage of pregnancy, reporting contractions and recent bloody vaginal discharge

****

Ms. Y is a G2 P1 who comes to you today in the birthing clinic. You know from her previous visits that she is 39 weeks AOG by LMP. 4 hours ago, she noted a mucoid, blood-tinged discharge of about two teaspoons from her vagina. She did not observe any watery discharge or bright red blood per vagina. Her abdominal cramping started this morning and occurred once every 8 minutes and has steadily increased in intensity and frequency to about once every 3-4 minutes. She reports that there is no decrease in fetal movement.

She went to 4 prenatal check-ups with you and had normal blood pressures, weight and a complete blood count 1 month prior to this visit. Today, Mrs. Y. denies any fever, cough/colds, excessive weight gain, epigastric pain or dysuria in her last month of pregnancy but claims that her feet have begun to swell over the past 2 months.

****

She is not aware of any history of anemia, bleeding disorder, hypertension, diabetes or asthma in her family.

Ms. Y is currently residing with her live-in partner of 2 years. She is a recent college graduate who works in a fast food service restaurant. She previously smoked in her teens but stopped 5 years ago, she denies consuming alcohol and use of illicit substances. She admits to be picky with her food, consuming more meat than vegetables and fish.

****

Physical examination:

BP 120/80 mm Hg	HR 92 beats/min	RR 16 breaths/min	T 37.1 ^{o}C	Weight 72 kg	Height 152 cm

General: Patient is conscious, coherent, not in distress.

HEENT: Pink conjunctivae, anicteric sclera, no anterior neck mass or cervical lymphadenopathy was noted.

Chest/lungs: Chest findings were normal.

Cardiac: Heart findings are also unremarkable.

Extremities: Pink nail beds, grade 1+ peripheral edema bilaterally

Abdominal exam or external exam of the fetus: Maternal bowel sounds are normal without guarding or organomegaly. Fundic height is 33 cm from the pubis. Leopold's maneuver suggests a single longitudinally oriented fetus, fetal back to the right, with cephalic presentation. The fetal head is not engaged in the pelvic brim. Estimated fetal weight is 2800 to 3000 grams. The fetal heart rate is 150 beats/min best heard at the right lower quadrant.

Internal exam: Pelvic examination confirms cephalic presentation. The cervix is 4 cm dilated and 75% effaced. Fetal station is -2 cm (from ischial spines). No bleeding is noted. Bag of waters is intact.

PLEASE ANSWER THE QUESTION <u>ON THE NEXT PAGE</u>.

PLEASE ANSWER THE FOLLOWING QUESTIONS. DO NOT MOVE AHEAD TO THE NEXT PART OF THE VIGNETTE UNTIL YOU HAVE ANSWERED THE QUESTIONS ON THIS PAGE.

Question5. *What are the* ***information in the patient's history and physical examination that are important for determining this diagnosis, order laboratory tests and subsequent therapy****?*
Please list them down including findings that are absent in the case (pertinent negatives), no need for explanation. (example: it is necessary to know the age of the patient)

Question 6.

At this point, what laboratory tests (if any), imaging studies and other tests or procedures would you request?

IMPORTANT: PLACE ANSWER SHEET IN ENVELOPE OR GIVE THE ANSWER SHEET TO AN EVALUATOR BEFORE CONTINUING.

CONTINUE READING THE VIGNETTE AND ANSWER **QUESTIONS 7a, 7b & 7c**IN **5 MINUTES**

Ms Y. is a 28-year old woman, obviously in the late stage of pregnancy, reporting contractions and recent bloody vaginal discharge

****

Ms. Y is a G2 P1 who comes to you today in the birthing clinic. You know from her previous visits that she is 39 weeks AOG by LMP. 4 hours ago, she noted a mucoid, blood-tinged discharge of about two teaspoons from her vagina. She did not observe any watery discharge or bright red blood per vagina. Her abdominal cramping started this morning and occurred once every 8 minutes and has steadily increased in intensity and frequency to about once every 3-4 minutes. She reports that there is no decrease in fetal movement.

She went to 4 prenatal check-ups with you and had normal blood pressures, weight and a complete blood count 1 month prior to this visit. Today, Mrs. Y. denies any fever, cough/colds, excessive weight gain, epigastric pain or dysuria in her last month of pregnancy but claims that her feet have begun to swell over the past 2 months.

****

She is not aware of any history of anemia, bleeding disorder, hypertension, diabetes or asthma in her family.
Ms. Y is currently residing with her live-in partner of 2 years. She is a recent college graduate who works in a fast food service restaurant. She previously smoked in her teens but stopped 5 years ago, she denies consuming alcohol and use of illicit substances. She admits to be picky with her food, consuming more meat than vegetables and fish.

****

Physical examination:

BP 120/80 mm Hg	HR 92 beats/min	RR 16 breaths/min	T 37.1 ^{o}C	Weight 72 kg	Height 152 cm

General: Patient is conscious, coherent, not in distress.
HEENT: Pink conjunctivae, anicteric sclera, no anterior neck mass or cervical lymphadenopathy was noted.
Chest/lungs: Chest findings were normal.
Cardiac: Heart findings are also unremarkable.
Extremities: Pink nail beds, grade 1+ peripheral edema bilaterally
Abdominal exam or external exam of the fetus: Maternal bowel sounds are normal without guarding or organomegaly. Fundic height is 33 cm from the pubis. Leopold's maneuver suggests a single longitudinally oriented fetus, fetal back to the right, with cephalic presentation. The fetal head is not engaged in the pelvic brim. Estimated fetal weight is 2800 to 3000 grams. The fetal heart rate is 150 beats/min best heard at the right lower quadrant.
Internal exam: Pelvic examination confirms cephalic presentation. The cervix is 4 cm dilated and 75% effaced. Fetal station is -2 cm (from ischial spines). No bleeding is noted. Bag of waters is intact.

****

Results of the laboratory tests done are as follows:
CBC: Hematocrit 0.38 hemoglobin 10.8 mg/dl WBC 9.8 with no bands. Platelets 190
Blood typing: A positive
Serum Chemistry: Creatinine 78 umol/L (normal 50-90 umol/L), BUN 4.8 mmol/L (normal 2.5-8 mmol/L)
Prothrombin time: INR 1.02 (normal 0.8 – 1.1), activity 97%
Partial thromboplastin time: Control: 22 seconds, patient: 20 seconds (Normal: 18-28 seconds)
Chest x-ray: No significant findings
12L ECG: sinus rhythm, normal axis

Clinical Course:

You decide to start NSS 1L + 20 units oxytocin to run for 6 hours (2mU/min).

On your labor watch you noted the following:

Time	Contractions	Fetal Heart Tones	Cervical Dilatation, Effacement	Fetal Station	Remarks
1 pm	Moderate-strong, 45 sec every 3-4 minutes	150	4cm 75% effaced	-2	Stable vital signs
4 pm	Strong, 50 sec, every 3 min	158	6cm 90% effaced	-1	No vaginal bleeding
6 pm	Strong, 50 sec, every 3 min	146	Full	0	BOW ruptured, clear
7 pm	Strong, 50 sec, every 3 min	142	Full	0	Stable vitals, patient pushing, clear amniotic fluid
8 pm	Strong, 50 sec, every 2 min	110	Full	0	Caput palpable, deceleration noted after contraction

PLEASE ANSWER THE QUESTION ON THE NEXT PAGE.

ANSWER **QUESTIONS 7a, 7b, 7c** IN **5 MINUTES**

PLEASE ANSWER THE FOLLOWING QUESTION. DO NOT MOVE AHEAD TO THE NEXT PART OF THE VIGNETTE UNTIL YOU HAVE ANSWERED THE QUESTION ON THIS PAGE.

Question 7a.

At this point, what is your primary diagnosis?

Question 7b.

What is the severity/urgency?

Question 7c.

What are key factors in this patient's case that helped you form your diagnosis and decide on the severity?

MPORTANT: PLACE ANSWER SHEET IN ENVELOPE OR GIVE THE ANSWER SHEET TO AN EVALUATOR BEFORE CONTINUING.

ANSWER **QUESTION 8** IN **4 MINUTES**

PLEASE ANSWER THE FOLLOWING QUESTIONS. DO NOT MOVE AHEAD TO THE NEXT PART OF THE VIGNETTE UNTIL YOU HAVE ANSWERED THE QUESTION ON THIS PAGE.

Question 8.

What would be your treatment plan?

MAKE SURE TO LIST YOUR INTERVENTIONS IN ORDER OF PRIORITY.

Please include pharmacologic and non pharmacologic interventions as deemed appropriate. Be specific with the generic name of the drug(s) that you would recommend. Include specific instructions if any to the patient. Include referrals you plan to make.

IMPORTANT: PLACE ANSWER SHEET IN ENVELOPE OR GIVE THE ANSWER SHEET TO AN EVALUATOR BEFORE CONTINUING.

CONTINUE READING THE VIGNETTE ANSWER **QUESTION 9** IN **4 MINUTES**

The patient underwent a primary Cesarean Section under regional anesthesia. There were no intra operative complications noted. The baby was also fine, with an Apgar score of 9 becoming 9.

Both the mother and the baby's post operative stay in the hospital were unremarkable.

On the 2nd day post op, the mother had her 1st bowel movement. Abdominal pain was minimal, and operative wound was clean and dry. Urine output was clear and adequate.

****

PLEASE ANSWER THE QUESTION <u>ON THE NEXT PAGE</u>.

PLEASE ANSWER THE FOLLOWING QUESTION. DO NOT MOVE AHEAD TO THE NEXT PART UNTIL YOU HAVE ANSWERED THE QUESTION ON THIS PAGE.

Question 9.

What 8-10 important items/steps would you include in managing the ***immediate postpartum period until before discharge****?*

Include pharmacologic and non-pharmacologic interventions and counseling.

IMPORTANT: PLACE ANSWER SHEET IN ENVELOPE OR GIVE THE ANSWER SHEET TO AN EVALUATOR BEFORE CONTINUING.

CPV #2: Post-partum Hemorrhage

FIRST DO NO HARM
CLINICAL PERFORMANCE VIGNETTE #2
February 2014

A. SAMPLING INFORMATION

A.1.	Date completed:	___ ___ /___ ___ /___ ___ (mm dd yy)	
A.2.	Time started:	__ __ : __ __ H	
A.3.	Time completed:	__ __ : __ __ H	
A.4.	Round:	0 1	
A.5.	City:	Quezon City	
A.6.	District:	_________________	
A.7.	Facility ID #:	____ ____ (Reg) ____ ____ (Prov) ____ ____ (Dist) ____ ____ ____ (Facility Code)	
A.8.	Provider ID #:	____ ____ (Reg) ____ ____ (Prov) ____ ____ (Dist) ______ (Pub/Pvt) ___ ___ ___ (Facility Code) ___ ___ ___ (Provider Code)	
A.9.	Provider type:	General Practitioner...	1
		Obstetrician-Gynecologist...	2
		Nurse-midwife..	3
		Nurse..	4
		Midwife...	5
		TBA/ *hilot*..	6
		Other, specify _____________...	7
A.10.	Age from last birthday:	___ ___	
A.11.	Sex:	Male..	1
		Female...	0
A.12.	Contact number *(landline and/or mobile)*:	*Landline number:* 0 ___ ___ (Area code) - ___ ___ ___ ___ ___ ___ ___	*Mobile number:* 0 9 ___ ___ - ___ ___ ___ ___ ___ ___ ___

CPV Administered by: ____
Signature over printed name

Admin. Code: [ ][ ][ ]

SUPERVISOR [ ][ ]

_________________________ _______________
Printed name and signature *Date*

SURVEY LEADER [ ][ ]

_________________________ _______________
Printed name and signature *Date*

Provider ID #	___ ___	___ ___	___ ___	_____	___ ___ ___	___ ___ ___
	Reg	*Prov*	*Dist*	*Pub/Pvt*	*Facility Code*	*Provider Code*

B. CONSENT

Dear Respondent,

We are from QURE Healthcare, a research group working with the Philippine Department of Health. With their endorsement, we are studying the clinical and economic consequences of referrals and outcomes, on parturient mothers in Quezon City. The results of this study, which are generalizable to other low to middle income countries, will be used to increase knowledge, build collaborative efforts locally and disseminate these findings with the World Bank, which is looking to fund policy initiatives to improve quality of care.

The information that you will provide today will be treated with utmost confidentiality. The data will be used for research purposes only. Your name or address and other personal information will be deleted from the questionnaire and only a code or number will connect your name with your answers.

The vignette takes approximately 30 minutes to complete. Your participation is voluntary. If you have any questions, you can ask me or can contact our research leads in the Philippines namely Dr Diana Tamondong-Lachica at 0922-8721686 and Ms Jenifer Tiu at 0928-5000122 or at (02) 9279686 loc 322.

Your signature indicates that you understood the purpose and mechanics of this study and that you are willing to participate.

____
Signature over printed name of respondent

Date: __ __ /__ __ /__ __
mm dd yy

____
Signature over printed name of enumerator

Date: __ __ /__ __ /__ __
mm dd yy

Did provider:	Consent and sign?	1
	Consent but not sign?	2
	Refuse to participate?	3

C. INSTRUCTIONS FOR COMPLETING THE VIGNETTE OR SIMULATED CASE:

Please consider the following case. It is meant to be typical of a case you would encounter in your practice.

Please bear in mind the following assumptions / conditions while answering the case:

1. Please assume that you are the only health professional handling the case;
2. Please assume that the setting and resources available of the case are identical to where you are currently / usually working;
3. Please handle the case as you would an actual case;
4. Expected answers that are not documented (written down) are considered not done / not thought of by the respondent.

The case takes approximately 30-35 minutes to complete. It is separated into parts: once you have read the clinical material on the particular section, complete the question on the answer sheets provided.

Once you have answered the questions, it is important that you detach/separate the answer sheet, put them inside the envelope provided, or give them to the interviewer /evaluator before proceeding to the next part of the case.

Please continue until all pages have been completed and the answer sheets placed in the envelope.

Your responses will remain confidential and the anonymity of the collected results is assured. We appreciate your participation. If you have any questions, please let us know.

READ THIS VIGNETTE AND ANSWER **QUESTION 1** IN **4 MINUTES**

Mrs. E.G., a 33-year-old is in the delivery room having just vaginally delivered a 3600 gram boy. She is bleeding and you are asked to evaluate her.

****

PLEASE ANSWER QUESTION 1 ON THE NEXT PAGE.

Provider ID # ____ ____ ____ ____ ____ ____ ______ ____ ____ ____ ____ ____ ____

Reg	Prov	Dist	Pub/Pvt	Facility Code	Provider Code

PLEASE ANSWER THE FOLLOWING QUESTION. DO NOT MOVE AHEAD TO THE NEXT PART OF THE VIGNETTE UNTIL YOU HAVE ANSWERED THE QUESTION ON THIS PAGE.

Question 1.

What are the 8-12 most important questions (information) that you want to ask from the ***patient's clinical history****? (Please be specific.)*

IMPORTANT: PLACE ANSWER SHEET IN ENVELOPE OR GIVE THE ANSWER SHEET TO AN EVALUATOR BEFORE CONTINUING.

CONTINUE READING THE VIGNETTE AND ANSWER **QUESTIONS 2 & 3** IN **4 MINUTES**

Mrs. E.G., a 33-year-old is in the delivery room having just vaginally delivered a 3600 gram boy. She is bleeding and you are asked to evaluate her.

Mrs. E.G., G4 now P4, came to your facility in active labor, 6 hours ago at 37 4/7 weeks age of gestation by last menstrual period. She had had regular prenatal check-ups with the midwife at a nearby lying-in clinic. There were no complications reported throughout her pregnancy including hypertension, diabetes and history of spotting or pre-term labor. She has no previous history of obstetrical complications with her prior 3 deliveries and her other 3 children are healthy without evidence of any developmental delay.

She gave birth four hours later to a live baby boy, 41 weeks by pediatric aging, APGAR scores were 9/9. No episiotomy was done. During the third stage of labor, cord traction was applied by the birth attendant, and the placenta was delivered 15 minutes later. Her intrapartum CBC showed: a hematocrit 0.35 (Normal 0.38-0.47); a hemoglobin 10.1 gm/dL (Normal 12-16 g/dl), microcytic, hypochromic RBCs, WBC 8.0 (Normal is 4.8 to 10.2) Platelets 310 (normal 150-450).

Mrs. E.G. denies bleeding disorders or bleeding prior to the onset of labor and reported to have had watery vaginal discharge. She also did not have any fever, cough/colds, excessive weight gain, epigastric pain or dysuria in her last month of pregnancy.

Mrs. E.G.'s previous pregnancies were all delivered vaginally and there was no history of hypertension, diabetes, hemorrhage or infection in any of her earlier pregnancies either.

****

PLEASE ANSWER THE QUESTION <u>ON THE NEXT PAGE</u>.

Provider ID # ___ ___ ___ ___ ___ ___ ______ ___ ___ ___ ___ ___ ___
Reg Prov Dist Pub/Pvt Facility Code Provider Code

PLEASE ANSWER THE FOLLOWING QUESTION. DO NOT MOVE AHEAD TO THE NEXT PART OF THE VIGNETTE UNTIL YOU HAVE ANSWERED THE QUESTION ON THIS PAGE.

Question 2

What are the most important questions you want to know about the patient's ***past medical history including OB-GYN history and associated medical illnesses****? (Please list.)*

Question 3.

What are the 9 to 11 most important questions you want to know about the ***family medical history and social history****? (Please list.)*

IMPORTANT: PLACE ANSWER SHEET IN ENVELOPE OR GIVE THE ANSWER SHEET TO AN EVALUATOR BEFORE CONTINUING.

CONTINUE READING THE VIGNETTE AND ANSWER **QUESTION 4** IN **4 MINUTES**

Mrs. E.G., a 33-year-old is in the delivery room having just vaginally delivered a 3600 gram boy. She is bleeding and you are asked to evaluate her.

****

Mrs. E.G., G4 now P4, came to your facility in active labor, 6 hours ago at 37 4/7 weeks age of gestation by last menstrual period. She had had regular prenatal check-ups with the midwife at a nearby lying-in clinic. There were no complications reported throughout her pregnancy including hypertension, diabetes and history of spotting or pre-term labor. She has no previous history of obstetrical complications with her prior 3 deliveries and her other 3 children are healthy without evidence of any developmental delay.

She gave birth four hours later to a live baby boy, 41 weeks by pediatric aging, APGAR scores were 9/9. No episiotomy was done. During the third stage of labor, cord traction was applied by the birth attendant, and the placenta was delivered 15 minutes later. Her intrapartum CBC showed: a hematocrit 0.35 (Normal 0.38-0.47); a hemoglobin 10.1 gm/dL (Normal 12-16 g/dl), microcytic, hypochromic RBCs, WBC 8.0 (Normal is 4.8 to 10.2) Platelets 310 (normal 150-450).

Mrs. E.G. denies bleeding disorders or bleeding prior to the onset of labor and reported to have had watery vaginal discharge. She also did not have any fever, cough/colds, excessive weight gain, epigastric pain or dysuria in her last month of pregnancy.

Mrs. E.G.'s previous pregnancies were all delivered vaginally and there was no history of hypertension, diabetes, hemorrhage or infection in any of her earlier pregnancies either.

****

She reports she has always been anemic and had intermittently taken iron supplements since she was a teenager. She has had no other surgeries. She is updated in her immunizations, including tetanus and hepatitis B. She has no known drug allergies. She recalls that she has regular menses prior to this pregnancy and has never used any form of contraception.

She is not aware of any history of anemia, bleeding disorder, hypertension, diabetes or asthma in her family.

Mrs. E.G. is married and lives with her husband of 10 years and their children. She works for a garment factory where she walks around 6 blocks daily to and from her house (except in the last 3 weeks of pregnancy). She does not smoke or drink alcohol and denies use of illicit substances. She eats a regular diet with no food preferences.

PLEASE ANSWER THE QUESTIONS ON THE NEXT PAGE.

Question 4.

What are the 6 to 8 most important elements of the ***physical examination*** *that need to be performed on this patient?*

(Note to examinee: ***Please be specific****. For example, do not say you would “examine the knee.” Instead, report what you would look for when you examine the knee, e.g. “examine the knee for redness, swelling, and point tenderness” or “evaluated knee for ligaments laxity and range of motion.”)*

IMPORTANT: PLACE ANSWER SHEET IN ENVELOPE OR GIVE THE ANSWER SHEET TO AN EVALUATOR BEFORE CONTINUING.

CONTINUE READING THE VIGNETTE AND ANSWER **QUESTIONS 5 & 6** IN **5 MINUTES**

Mrs. E.G., a 33-year-old is in the delivery room having just vaginally delivered a 3600 gram boy. She is bleeding and you are asked to evaluate her.

****

Mrs. E.G., G4 now P4, came to your facility in active labor, 6 hours ago at 37 4/7 weeks age of gestation by last menstrual period. She had had regular prenatal check-ups with the midwife at a nearby lying-in clinic. There were no complications reported throughout her pregnancy including hypertension, diabetes and history of spotting or pre-term labor. She has no previous history of obstetrical complications with her prior 3 deliveries and her other 3 children are healthy without evidence of any developmental delay.

She gave birth four hours later to a live baby boy, 41 weeks by pediatric aging, APGAR scores were 9/9. No episiotomy was done. During the third stage of labor, cord traction was applied by the birth attendant, and the placenta was delivered 15 minutes later. Her intrapartum CBC showed: a hematocrit 0.35 (Normal 0.38-0.47); a hemoglobin 10.1 gm/dL (Normal 12-16 g/dl), microcytic, hypochromic RBCs, WBC 8.0 (Normal is 4.8 to 10.2) Platelets 310 (normal 150-450).

Mrs. E.G. denies bleeding disorders or bleeding prior to the onset of labor and reported to have had watery vaginal discharge. She also did not have any fever, cough/colds, excessive weight gain, epigastric pain or dysuria in her last month of pregnancy.

Mrs. E.G.'s previous pregnancies were all delivered vaginally and there was no history of hypertension, diabetes, hemorrhage or infection in any of her earlier pregnancies either.

****

She reports she has always been anemic and had intermittently taken iron supplements since she was a teenager. She has had no other surgeries. She is updated in her immunizations, including tetanus and hepatitis B. She has no known drug allergies. She recalls that she has regular menses prior to this pregnancy and has never used any form of contraception.

She is not aware of any history of anemia, bleeding disorder, hypertension, diabetes or asthma in her family.

Mrs. E.G. is married and lives with her husband of 10 years and their children. She works for a garment factory where she walks around 6 blocks daily to and from her house (except in the last 3 weeks of pregnancy). She does not smoke or drink alcohol and denies use of illicit substances. She eats a regular diet with no food preferences.

****

Physical examination:

BP 95/50 mm Hg	HR 114 beats/min	RR 16 breaths/min	T 37.0 ^{o}C	Weight 72 kg	Height 154 cm

General: Patient is conscious, coherent, not in distress.
HEENT: She has slightly pale conjunctivae and anicteric sclerae. No anterior neck mass was noted. She has no cervical lymphadenopathy.
Chest/lungs: Chest findings were normal.
Cardiac: Heart findings are also unremarkable except for tachycardia.
Extremities: Slightly cold to the touch, no edema
Abdominal: Enlarged uterus that is boggy to palpation, no tenderness noted
Internal exam: Brisk bleeding noted from the cervical os. No evidence of cervical or vaginal lacerations and frank blood clots
The placenta has been removed and appears grossly intact

PLEASE ANSWER THE QUESTION <u>ON THE NEXT PAGE</u>.

PLEASE ANSWER THE FOLLOWING QUESTIONS. DO NOT MOVE AHEAD TO THE NEXT PART OF THE VIGNETTE UNTIL YOU HAVE ANSWERED THE QUESTIONS ON THIS PAGE.

Question5. *What are the* ***information in the patient's history and physical examination that are important for determining this diagnosis, order laboratory tests and subsequent therapy****?*
Please list them down including findings that are absent in the case (pertinent negatives), no need for explanation. (example: it is necessary to know the age of the patient)

Question 6.

At this point, what laboratory tests (if any), imaging studies and other tests or procedures would you request?

IMPORTANT: PLACE ANSWER SHEET IN ENVELOPE OR GIVE THE ANSWER SHEET TO AN EVALUATOR BEFORE CONTINUING.

CONTINUE READING THE VIGNETTE AND ANSWER **QUESTIONS 7a, 7b & 7c**IN **5 MINUTES**

Mrs. E.G., a 33-year-old is in the delivery room having just vaginally delivered a 3600 gram boy. She is bleeding and you are asked to evaluate her.

****

Mrs. E.G., G4 now P4, came to your facility in active labor, 6 hours ago at 37 4/7 weeks age of gestation by last menstrual period. She had had regular prenatal check-ups with the midwife at a nearby lying-in clinic. There were no complications reported throughout her pregnancy including hypertension, diabetes and history of spotting or pre-term labor. She has no previous history of obstetrical complications with her prior 3 deliveries and her other 3 children are healthy without evidence of any developmental delay.

She gave birth four hours later to a live baby boy, 41 weeks by pediatric aging, APGAR scores were 9/9. No episiotomy was done. During the third stage of labor, cord traction was applied by the birth attendant, and the placenta was delivered 15 minutes later. Her intrapartum CBC showed: a hematocrit 0.35 (Normal 0.38-0.47); a hemoglobin 10.1 gm/dL (Normal 12-16 g/dl), microcytic, hypochromic RBCs, WBC 8.0 (Normal is 4.8 to 10.2) Platelets 310 (normal 150-450).

Mrs. E.G. denies bleeding disorders or bleeding prior to the onset of labor and reported to have had watery vaginal discharge. She also did not have any fever, cough/colds, excessive weight gain, epigastric pain or dysuria in her last month of pregnancy.

Mrs. E.G.'s previous pregnancies were all delivered vaginally and there was no history of hypertension, diabetes, hemorrhage or infection in any of her earlier pregnancies either.

****

She reports she has always been anemic and had intermittently taken iron supplements since she was a teenager. She has had no other surgeries. She is updated in her immunizations, including tetanus and hepatitis B. She has no known drug allergies. She recalls that she has regular menses prior to this pregnancy and has never used any form of contraception.

She is not aware of any history of anemia, bleeding disorder, hypertension, diabetes or asthma in her family.

Mrs. E.G. is married and lives with her husband of 10 years and their children. She works for a garment factory where she walks around 6 blocks daily to and from her house (except in the last 3 weeks of pregnancy). She does not smoke or drink alcohol and denies use of illicit substances. She eats a regular diet with no food preferences.

****

Physical examination:

BP 95/50 mm Hg	HR 114 beats/min	RR 16 breaths/min	T 37.0 °C	Weight 72 kg	Height 154 cm

General: Patient is conscious, coherent, not in distress.
HEENT: She has slightly pale conjunctivae and anicteric sclerae. No anterior neck mass was noted. She has no cervical lymphadenopathy.
Chest/lungs: Chest findings were normal.
Cardiac: Heart findings are also unremarkable except for tachycardia.
Extremities: Slightly cold to the touch, no edema
Abdominal: Enlarged uterus that is boggy to palpation, no tenderness noted
Internal exam: Brisk bleeding noted from the cervical os. No evidence of cervical or vaginal lacerations and frank blood clots
The placenta has been removed and appears grossly intact

****

Results of the laboratory tests done are as follows:

Repeat CBC: Hematocrit 0.25 hemoglobin 8.8 mg/dL. WBC 9.8 with no bands. Platelets 190

Blood typing and crossmatch 2U PRBC: A positive

Prothrombin time: INR 1.02 (normal 0.8 – 1.1), activity 97%

Partial thromboplastin time: Control: 22 seconds, patient: 20 seconds (Normal: 18-28 seconds)

Liver enzymes: AST 46 IU/L (11-47 IU/L) ALT 30 IU/L (7-53 IU/L)

Serum Chemistry: Creatinine 78 umol/L (normal 50-90 umol/L), BUN 4.8 mmol/L (normal 2.5-8 mmol/L), sodium 145 mmol/L (normal 135-145 mmol/L), potassium 3.7 mmol/L (normal 3.6-5.1 mmol/L), calcium 2.38 mmol/L (normal 2.18-2.58 mmol/L), magnesium 0.90 mmol/L (normal 0.75-0.95 mmol/L)

Fibrinogen: Normal

Urinalysis: Normal

12L ECG: Sinus tachycardia, normal axis

Chest x-ray: Normal

Bedside pelvic ultrasound*:* Enlarged uterus with intrauterine placental fragments, no intra-abdominal fluid noted.

Pelvic CT scan: Enlarged uterus with intrauterine placental fragments, no intraabdominal fluid noted.

PLEASE ANSWER THE QUESTION <u>ON THE NEXT PAGE</u>.

ANSWER **QUESTIONS 7a, 7b, 7c** IN **5 MINUTES**

PLEASE ANSWER THE FOLLOWING QUESTION. DO NOT MOVE AHEAD TO THE NEXT PART OF THE VIGNETTE UNTIL YOU HAVE ANSWERED THE QUESTION ON THIS PAGE.

Question 7a.

At this point, what is your primary diagnosis?

Question 7b.

What is the severity/urgency?

Question 7c.

What are key factors in this patient's case that helped you form your diagnosis and decide on the severity?

MPORTANT: PLACE ANSWER SHEET IN ENVELOPE OR GIVE THE ANSWER SHEET TO AN EVALUATOR BEFORE CONTINUING.

Provider ID # ___ ___ ___ ___ ___ ___ ___ ___ ___ ___ ___ ___ ___ ___

Reg | Prov | Dist | Pub/Pvt | Facility Code | Provider Code

ANSWER **QUESTION 8** IN **4 MINUTES**

PLEASE ANSWER THE FOLLOWING QUESTIONS. DO NOT MOVE AHEAD TO THE NEXT PART OF THE VIGNETTE UNTIL YOU HAVE ANSWERED THE QUESTION ON THIS PAGE.

Question 8.

What would be your treatment plan?

MAKE SURE TO LIST YOUR INTERVENTIONS IN ORDER OF PRIORITY.

Please include pharmacologic and non pharmacologic interventions as deemed appropriate. Be specific with the generic name of the drug(s) that you would recommend. Include specific instructions if any to the patient. Include referrals you plan to make.

IMPORTANT: PLACE ANSWER SHEET IN ENVELOPE OR GIVE THE ANSWER SHEET TO AN EVALUATOR BEFORE CONTINUING.

CONTINUE READING THE VIGNETTE ANSWER **QUESTION 9** IN **4 MINUTES**

Clinical Course:

Her vital signs are as follows: BP of 90/50 mm Hg, heart rate of 120/minute, respiratory rate of 16/minute. She is drowsy but arousable and follows commands. She is flat on bed, with clear breath sounds. Extremities are still slightly cold and clammy.

Attempts at manual extraction of the placental fragments were unsuccessful. Bleeding per vagina persists and is unchanged although the uterus is no longer boggy. Direct visualization of the cervical os shows no lacerations. The repeat CBC shows hemoglobin level of 72 g/L, and hematocrit of 0.23.

****

PLEASE ANSWER THE QUESTION ON THE NEXT PAGE.

ANSWER **QUESTION 9** IN **4 MINUTES**

PLEASE ANSWER THE FOLLOWING QUESTIONS. DO NOT MOVE AHEAD TO THE NEXT PART OF THE VIGNETTE UNTIL YOU HAVE ANSWERED THE QUESTION ON THIS PAGE.

Question 9.
What would be your next step/s given the clinical course of the patient?

IMPORTANT: PLACE ANSWER SHEET IN ENVELOPE OR GIVE THE ANSWER SHEET TO AN EVALUATOR BEFORE CONTINUING.

CONTINUE READING THE VIGNETTE ANSWER **QUESTION 10** IN **4 MINUTES**

Clinical Course:

Dilatation and curettage was done under local anesthesia. Around 80cc of retained placental fragments were evacuated. No significant bleeding per vagina was noted while the uterus is now contracted.

Repeat vital signs showed BP 100/60 HR 100 RR 14 afebrile. She appears comfortable and breath sounds are clear.

Total of 2 units whole blood and 1 unit of packed RBC have been transfused with 1 unit pRBC ongoing transfusion.

****

PLEASE ANSWER THE QUESTION <u>ON THE NEXT PAGE</u>.

PLEASE ANSWER THE FOLLOWING QUESTION. DO NOT MOVE AHEAD TO THE NEXT PART UNTIL YOU HAVE ANSWERED THE QUESTION ON THIS PAGE.

Question 10

What 8-10 important items/steps would you include in managing the ***immediate postpartum period until before discharge****?*

Include pharmacologic and non-pharmacologic interventions and counseling.

IMPORTANT: PLACE ANSWER SHEET IN ENVELOPE OR GIVE THE ANSWER SHEET TO AN EVALUATOR BEFORE CONTINUING.

CPV #3: Pre-eclampsia

FIRST DO NO HARM
CLINICAL PERFORMANCE VIGNETTE #3
February 2014

A. SAMPLING INFORMATION

A.1.	Date completed:	___ ___ /___ ___ /___ ___ *mm dd yy*	
A.2.	Time started:	__ __ : __ __ H	
A.3.	Time completed:	__ __ : __ __ H	
A.4.	Round:	0 1	
A.5.	City:	Quezon City	
A.6.	District:	________________	
A.7.	Facility ID #:	____ ____ ____ ____ ____ ____ ____ ____ ____ *Reg Prov Dist Facility Code*	
A.8.	Provider ID #:	____ ____ ____ ____ ____ ____ ______ ___ ___ ___ ___ ___ ___ *Reg Prov Dist Pub/Pvt Facility Code Provider Code*	
A.9.	Provider type:	General Practitioner..	1
		Obstetrician-Gynecologist...	2
		Nurse-midwife..	3
		Nurse...	4
		Midwife..	5
		TBA/ *hilot*..	6
		Other, specify _____________..	7
A.10.	Age from last birthday:	___ ___	
A.11.	Sex:	Male..	1
		Female..	0
A.12.	Contact number *(landline and/or mobile)*:	*Landline number:* 0 ___ ___ - ___ ___ ___ ___ ___ ___ ___ Area code	*Mobile number:* 0 9 ___ ___ - ___ ___ ___ ___ ___ ___ ___

CPV Administered by: ____
Signature over printed name

Admin. Code: [ ] [ ] [ ]

SUPERVISOR [ ] [ ]	SURVEY LEADER [ ] [ ]
______________________ ____________ *Printed name and signature* *Date*	______________________ ____________ *Printed name and signature* *Date*

B. CONSENT

Dear Respondent,

We are from QURE Healthcare, a research group working with the Philippine Department of Health. With their endorsement, we are studying the clinical and economic consequences of referrals and outcomes, on parturient mothers in Quezon City. The results of this study, which are generalizable to other low to middle income countries, will be used to increase knowledge, build collaborative efforts locally and disseminate these findings with the World Bank, which is looking to fund policy initiatives to improve quality of care.

The information that you will provide today will be treated with utmost confidentiality. The data will be used for research purposes only. Your name or address and other personal information will be deleted from the questionnaire and only a code or number will connect your name with your answers.

The vignette takes approximately 30 minutes to complete. Your participation is voluntary. If you have any questions, you can ask me or can contact our research leads in the Philippines namely Dr Diana Tamondong-Lachica at 0922-8721686 and Ms Jenifer Tiu at 0928-5000122 or at (02) 9279686 loc 322.

Your signature indicates that you understood the purpose and mechanics of this study and that you are willing to participate.

______________________________________ Signature over printed name of respondent

______________________________________ Signature over printed name of enumerator

Date: __ __ /__ __ /__ __
mm dd yy

Date: __ __ /__ __ /__ __
mm dd yy

Did provider:	Consent and sign?	1
	Consent but not sign?	2
	Refuse to participate?	3

C. INSTRUCTIONS FOR COMPLETING THE VIGNETTE OR SIMULATED CASE:

Please consider the following case. It is meant to be typical of a case you would encounter in your practice.

Please bear in mind the following assumptions / conditions while answering the case:

1. Please assume that you are the only health professional handling the case;
2. Please assume that the setting and resources available of the case are identical to where you are currently / usually working;
3. Please handle the case as you would an actual case;
4. Expected answers that are not documented (written down) are considered not done / not thought of by the respondent.

The case takes approximately 30-35 minutes to complete. It is separated into parts: once you have read the clinical material on the particular section, complete the question on the answer sheets provided.

Once you have answered the questions, it is important that you detach/separate the answer sheet, put them inside the envelope provided, or give them to the interviewer /evaluator before proceeding to the next part of the case.

Please continue until all pages have been completed and the answer sheets placed in the envelope.

Your responses will remain confidential and the anonymity of the collected results is assured. We appreciate your participation. If you have any questions, please let us know.

READ THIS VIGNETTE AND ANSWER **QUESTION 1** IN **4 MINUTES**

Mrs. P is a 22-year old woman who comes to your clinic for labor pains. You have been following her throughout her pregnancy.

****

PLEASE ANSWER QUESTION 1 ON THE NEXT PAGE.

Provider ID # ____ ____ ____ ____ ____ ____ ______ ____ ____ ____ ____ ____ ____

Reg | Prov | Dist | Pub/Pvt | Facility Code | Provider Code

PLEASE ANSWER THE FOLLOWING QUESTION. DO NOT MOVE AHEAD TO THE NEXT PART OF THE VIGNETTE UNTIL YOU HAVE ANSWERED THE QUESTION ON THIS PAGE.

Question 1.

*What are the 8-12 most important questions (information) that you want to ask from the **patient's clinical history**? (Please be specific.)*

IMPORTANT: PLACE ANSWER SHEET IN ENVELOPE OR GIVE THE ANSWER SHEET TO AN EVALUATOR BEFORE CONTINUING.

CONTINUE READING THE VIGNETTE AND ANSWER **QUESTIONS 2 & 3** IN **4 MINUTES**

Mrs. P is a 22-year old woman who comes to your clinic for labor pains. You have been following her throughout her pregnancy.

On history, you found out that Mrs. P's pregnancy is 38 and 6/7 weeks AOG by LMP.
This is her 1st pregnancy (G1P0) and she has had 2 prenatal check-ups with you, the last one was when she was 24 weeks AOG. Her vital signs have been normal and your latest estimate of fetal weight is appropriate for gestational age. Her hemoglobin is 12.8 g/dl, urine protein/glucose is negative and her 75g OGTT was normal.

She has been feeling irregular contractions in lower abdomen which often come with walking but stops with resting. She denies watery or bloody vaginal discharge. Two weeks ago she noted swelling in her ankles that she thinks has progressed.

She has no vaginal bleeding during her pregnancy. She reports no headache, blurring of vision, RUQ or epigastric pain, difficulty of breathing, or nausea and vomiting. Her vital signs in the clinic today are: Blood pressure 170/90, pulse rate 102, respiratory rate 18, temp 37.1^{o}C ; Weight 69 kg; and Height 152cm

****

PLEASE ANSWER THE QUESTION <u>ON THE NEXT PAGE</u>.

PLEASE ANSWER THE FOLLOWING QUESTION. DO NOT MOVE AHEAD TO THE NEXT PART OF THE VIGNETTE UNTIL YOU HAVE ANSWERED THE QUESTION ON THIS PAGE.

Question 2

*What are the most important questions you want to know about the patient's **past medical history including OB-GYN history and associated medical illnesses**? (Please list.)*

Question 3.

*What are the 9 to 11 most important questions you want to know about the **family medical history and social history**? (Please list.)*

IMPORTANT: PLACE ANSWER SHEET IN ENVELOPE OR GIVE THE ANSWER SHEET TO AN EVALUATOR BEFORE CONTINUING.

CONTINUE READING THE VIGNETTE AND ANSWER **QUESTION 4** IN **4 MINUTES**

Mrs. P is a 22-year old woman who comes to your clinic for labor pains. You have been following her throughout her pregnancy.

****

On history, you found out that Mrs. P's pregnancy is 38 and 6/7 weeks AOG by LMP.
This is her 1st pregnancy (G1P0) and she has had 2 prenatal check-ups with you, the last one was when she was 24 weeks AOG. Her vital signs have been normal and your latest estimate of fetal weight is appropriate for gestational age. Her hemoglobin is 12.8 g/dl, urine protein/glucose is negative and her 75g OGTT was normal.

She has been feeling irregular contractions in lower abdomen which often come with walking but stops with resting. She denies watery or bloody vaginal discharge. Two weeks ago she noted swelling in her ankles that she thinks has progressed.

She has no vaginal bleeding during her pregnancy. She reports no headache, blurring of vision, RUQ or epigastric pain, difficulty of breathing, or nausea and vomiting. Her vital signs in the clinic today are: Blood pressure 170/90, pulse rate 102, respiratory rate 18, temp 37.1^{o}C ; Weight 69 kg; and Height 152cm

She specifically has no history of diabetes or hypertension or bleeding disorders. She has not had any previous hospitalizations or surgeries. She denies any food or drug allergies. She is not taking any medicines except for Iron and Folic acid supplements. She recalls that she has regular menses prior to this pregnancy and has never used any form of contraception.

Her mother has diabetes while father has hypertension; they both take maintenance oral medications. She claims her mother did not have any problems during pregnancy and childbirth. She smokes around 1-3 sticks of cigarettes per day for the past 5 years but claims she has stopped since learning she was pregnant. She denies drinking alcoholic beverages or using illicit drugs.

PLEASE ANSWER THE QUESTIONS ON THE NEXT PAGE.

Question 4.

*What are the 6 to 8 most important elements of the **physical examination** that need to be performed on this patient?*

*(Note to examinee: **Please be specific**. For example, do not say you would "examine the knee." Instead, report what you would look for when you examine the knee, e.g. "examine the knee for redness, swelling, and point tenderness" or "evaluated knee for ligaments laxity and range of motion.")*

IMPORTANT: PLACE ANSWER SHEET IN ENVELOPE OR GIVE THE ANSWER SHEET TO AN EVALUATOR BEFORE CONTINUING.

CONTINUE READING THE VIGNETTE AND ANSWER **QUESTIONS 5** & 6 IN **5 MINUTES**

Mrs. P is a 22-year old woman who comes to your clinic for labor pains. You have been following her throughout her pregnancy.
****

On history, you found out that Mrs. P's pregnancy is 38 and 6/7 weeks AOG by LMP.
This is her 1st pregnancy (G1P0) and she has had 2 prenatal check-ups with you, the last one was when she was 24 weeks AOG. Her vital signs have been normal and your latest estimate of fetal weight is appropriate for gestational age. Her hemoglobin is 12.8 g/dl, urine protein/glucose is negative and her 75g OGTT was normal.

She has been feeling irregular contractions in lower abdomen which often come with walking but stops with resting. She denies watery or bloody vaginal discharge. Two weeks ago she noted swelling in her ankles that she thinks has progressed.

She has no vaginal bleeding during her pregnancy. She reports no headache, blurring of vision, RUQ or epigastric pain, difficulty of breathing, or nausea and vomiting. Her vital signs in the clinic today are: Blood pressure 170/90, pulse rate 102, respiratory rate 18, temp 37.1^{o}C ; Weight 69 kg; and Height 152cm

She specifically has no history of diabetes or hypertension or bleeding disorders. She has not had any previous hospitalizations or surgeries. She denies any food or drug allergies. She is not taking any medicines except for Iron and Folic acid supplements. She recalls that she has regular menses prior to this pregnancy and has never used any form of contraception.

Her mother has diabetes while father has hypertension; they both take maintenance oral medications. She claims her mother did not have any problems during pregnancy and childbirth. She smokes around 1-3 sticks of cigarettes per day for the past 5 years but claims she has stopped since learning she was pregnant. She denies drinking alcoholic beverages or using illicit drugs.
****

Physical examination:

Repeat vital signs after 30 minutes:

BP 150/100 mm Hg	HR 106 beats/min	RR 20 breaths/min	T 37.2 ^{o}C	Weight 62 kg	Height 149 cm

General: Patient is conscious, coherent, not in distress.

HEENT: She has pink conjunctivae and anicteric sclerae. No anterior neck mass was noted. She has no cervical lymphadenopathy.

Chest/lungs: Chest findings were normal.

Cardiac: Heart findings are also unremarkable except for a flow murmur heard best in the 2nd ICS on the L.

Extremities: There is 2+ bilateral lower leg edema half way to the knee.

Neurologic: No focal deficits

Abdominal: Normal maternal bowel sounds with guarding or organomegaly. There is no epigastric nor RUQ tenderness.
FHT - 146 beats per minute Abdomen was globular with fundal height of 37 cm.
Leopolds maneuver showed a fetus that is vertically oriented and cephalic in presentation.
Estimated fetal weight 2600 - 2800 gm

Internal exam: Normal external genitalia, nulliparous vagina, cervix is 1cm and 25% effaced, firm, posterior position, fetal station is +0

Pelvimetry: adequate clinically

Labor monitoring: Uterine contractions are moderate in intensity, irregular in frequency, every 5-10 minutes and lasting 5-10 seconds. No fetal bradycardia after maternal contractions were noted.

PLEASE ANSWER THE QUESTION ON THE NEXT PAGE.

PLEASE ANSWER THE FOLLOWING QUESTIONS. DO NOT MOVE AHEAD TO THE NEXT PART OF THE VIGNETTE UNTIL YOU HAVE ANSWERED THE QUESTIONS ON THIS PAGE.

Question5. *What are the* ***information in the patient's history and physical examination that are important for determining this diagnosis, order laboratory tests and subsequent therapy****?*
Please list them down including findings that are absent in the case (pertinent negatives), no need for explanation. (example: it is necessary to know the age of the patient)

Question 6.

At this point, what laboratory tests (if any), imaging studies and other tests or procedures would you request?

IMPORTANT: PLACE ANSWER SHEET IN ENVELOPE OR GIVE THE ANSWER SHEET TO AN EVALUATOR BEFORE CONTINUING.

CONTINUE READING THE VIGNETTE AND ANSWER **QUESTIONS 7a, 7b & 7c**IN **5 MINUTES**

Mrs. P is a 22-year old woman who comes to your clinic for labor pains. You have been following her throughout her pregnancy.
****

On history, you found out that Mrs. P's pregnancy is 38 and 6/7 weeks AOG by LMP.
This is her 1st pregnancy (G1P0) and she has had 2 prenatal check-ups with you, the last one was when she was 24 weeks AOG. Her vital signs have been normal and your latest estimate of fetal weight is appropriate for gestational age. Her hemoglobin is 12.8 g/dl, urine protein/glucose is negative and her 75g OGTT was normal.

She has been feeling irregular contractions in lower abdomen which often come with walking but stops with resting. She denies watery or bloody vaginal discharge. Two weeks ago she noted swelling in her ankles that she thinks has progressed.

She has no vaginal bleeding during her pregnancy. She reports no headache, blurring of vision, RUQ or epigastric pain, difficulty of breathing, or nausea and vomiting. Her vital signs in the clinic today are: Blood pressure 170/90, pulse rate 102, respiratory rate 18, temp 37.1^{o}C ; Weight 69 kg; and Height 152cm

She specifically has no history of diabetes or hypertension or bleeding disorders. She has not had any previous hospitalizations or surgeries. She denies any food or drug allergies. She is not taking any medicines except for Iron and Folic acid supplements. She recalls that she has regular menses prior to this pregnancy and has never used any form of contraception.

Her mother has diabetes while father has hypertension; they both take maintenance oral medications. She claims her mother did not have any problems during pregnancy and childbirth. She smokes around 1-3 sticks of cigarettes per day for the past 5 years but claims she has stopped since learning she was pregnant. She denies drinking alcoholic beverages or using illicit drugs.
****

Physical examination:
Repeat vital signs after 30 minutes:

BP 150/100 mm Hg	HR 106 beats/min	RR 20 breaths/min	T 37.2 ^{o}C	Weight 62 kg	Height 149 cm

General: Patient is conscious, coherent, not in distress.
HEENT: She has pink conjunctivae and anicteric sclerae. No anterior neck mass was noted. She has no cervical lymphadenopathy.
Chest/lungs: Chest findings were normal.
Cardiac: Heart findings are also unremarkable except for a flow murmur heard best in the 2nd ICS on the L.
Extremities: There is 2+ bilateral lower leg edema half way to the knee.
Neurologic: No focal deficits
Abdominal: Normal maternal bowel sounds with guarding or organomegaly. There is no epigastric nor RUQ tenderness.
FHT - 146 beats per minute Abdomen was globular with fundal height of 37 cm.
Leopolds maneuver showed a fetus that is vertically oriented and cephalic in presentation.
Estimated fetal weight 2600 - 2800 gm
Internal exam: Normal external genitalia, nulliparous vagina, cervix is 1cm and 25% effaced, firm, posterior position, fetal station is +0
Pelvimetry: adequate clinically
Labor monitoring: Uterine contractions are moderate in intensity, irregular in frequency, every 5-10 minutes and lasting 5-10 seconds. No fetal bradycardia after maternal contractions were noted.

****

Results of the laboratory tests done are as follows:

CBC: Hgb 11.5 mg/dl Hct 0.34 WBC 11,000 (0.75 seg, 0.24 lymph) Platelet 950,000

Urine albumin: 2+ (dipstick)

Liver enzymes: AST 122 IU/L (11-47 IU/L) ALT 184 IU/L (7-53 IU/L)

Creatinine: 60 umol/L (normal: 50-90 umol/L)

Lactate dehydrogenase (LDH): 450 U/L (normal: 115-221 U/L)

Liver enzymes 1.5 times

Coagulation: PT 12 sec (control: 14 sec) INR 0.95 PTT 28 sec (control: 25-38 sec)

Peripheral blood smear: normal, no schistocytes or signs of hemolysis seen

12L ECG: Sinus tachycardia, normal axis without evidence of ischemia

Chest x-ray: Normal

PLEASE ANSWER THE QUESTION <u>ON THE NEXT PAGE</u>.

Provider ID #	____ ____	____ ____	____ ____	______	____ ____ ____	____ ____ ____
	Reg	*Prov*	*Dist*	*Pub/Pvt*	*Facility Code*	*Provider Code*

ANSWER **QUESTIONS 7a, 7b, 7c** IN **5 MINUTES**

PLEASE ANSWER THE FOLLOWING QUESTION. DO NOT MOVE AHEAD TO THE NEXT PART OF THE VIGNETTE UNTIL YOU HAVE ANSWERED THE QUESTION ON THIS PAGE.

Question 7a.
At this point, what is your primary diagnosis?

Question 7b.
What is the severity/urgency?

Question 7c.
What are key factors in this patient's case that helped you form your diagnosis and decide on the severity?

MPORTANT: PLACE ANSWER SHEET IN ENVELOPE OR GIVE THE ANSWER SHEET TO AN EVALUATOR BEFORE CONTINUING.

ANSWER **QUESTION 8** IN **4 MINUTES**

PLEASE ANSWER THE FOLLOWING QUESTIONS. DO NOT MOVE AHEAD TO THE NEXT PART OF THE VIGNETTE UNTIL YOU HAVE ANSWERED THE QUESTION ON THIS PAGE.

Question 8.

What would be your treatment plan?
MAKE SURE TO LIST YOUR INTERVENTIONS IN ORDER OF PRIORITY.
Please include pharmacologic and non pharmacologic interventions as deemed appropriate. Be specific with the generic name of the drug(s) that you would recommend. Include specific instructions if any to the patient. Include referrals you plan to make.

IMPORTANT: PLACE ANSWER SHEET IN ENVELOPE OR GIVE THE ANSWER SHEET TO AN EVALUATOR BEFORE CONTINUING.

CONTINUE READING THE VIGNETTE ANSWER **QUESTION 9** IN **4 MINUTES**

Clinical Course:
The patient was prepped for delivery. She was wheeled into the labor room.

Hydralazine 10 mg IV every 20 minutes was given.

The patient's BP reading slowly decreased and later stabilized to 140/90 after the 3rd dose of hydralazine IV was given.

Magnesium sulfate 5 g IV (over 20 minutes) was also given. Maintenance of 1 g/h was also started.

After 8 hours, patient reached 10 cm cervical dilatation, station -2

Uterine contractions were strong and occurred every 40 seconds with no note of fetal bradycardia/distress

Maternal vital signs: BP 140/90 HR 100 RR 26

****

PLEASE ANSWER THE QUESTION <u>ON THE NEXT PAGE</u>.

ANSWER **QUESTION 9** IN **4 MINUTES**

PLEASE ANSWER THE FOLLOWING QUESTIONS. DO NOT MOVE AHEAD TO THE NEXT PART OF THE VIGNETTE UNTIL YOU HAVE ANSWERED THE QUESTION ON THIS PAGE.

Question 9.
What would be your next step/s given the clinical course of the patient?

IMPORTANT: PLACE ANSWER SHEET IN ENVELOPE OR GIVE THE ANSWER SHEET TO AN EVALUATOR BEFORE CONTINUING.

CONTINUE READING THE VIGNETTE ANSWER **QUESTION 10** IN **4 MINUTES**

Clinical Course:
Patient underwent vaginal delivery to a live baby boy, APGAR 9,9

She underwent repair of a first-degree perineal tear. The placenta is unremarkable and blood loss was only 150cc. She denies any symptoms.

Repeat vital signs: BP 120/80 HR 98 RR 14 Temp 37

Abdominal exam revealed a contracted uterus and there is only minimal blood per vaginal pad

****

PLEASE ANSWER THE QUESTION ON THE NEXT PAGE.

Provider ID # ____ ____ ____ ____ ____ ____ ______ ____ ____ ____ ____ ____ ____
Reg Prov Dist Pub/Pvt Facility Code Provider Code

PLEASE ANSWER THE FOLLOWING QUESTION. DO NOT MOVE AHEAD TO THE NEXT PART UNTIL YOU HAVE ANSWERED THE QUESTION ON THIS PAGE.

Question 10.

What 8-10 important items/steps would you include in managing the ***immediate postpartum period until before discharge****?*

Include pharmacologic and non-pharmacologic interventions and counseling.

IMPORTANT: PLACE ANSWER SHEET IN ENVELOPE OR GIVE THE ANSWER SHEET TO AN EVALUATOR BEFORE CONTINUING.
